# The Rat Genome Database (RGD) facilitates genomic and phenotypic data integration across multiple species for biomedical research

**DOI:** 10.1007/s00335-021-09932-x

**Published:** 2021-11-05

**Authors:** M. L. Kaldunski, J. R. Smith, G. T. Hayman, K. Brodie, J. L. De Pons, W. M. Demos, A. C. Gibson, M. L. Hill, M. J. Hoffman, L. Lamers, S. J. F. Laulederkind, H. S. Nalabolu, K. Thorat, J. Thota, M. Tutaj, M. A. Tutaj, M. Vedi, S. J. Wang, S. Zacher, M. R. Dwinell, A. E. Kwitek

**Affiliations:** 1 Department of Biomedical Engineering, The Rat Genome Database, Medical College of Wisconsin, Milwaukee, WI USA; 2 Department of Physiology, Medical College of Wisconsin, Milwaukee, WI USA; 3 Clinical and Translational Science Institute, Medical College of Wisconsin, Milwaukee, WI USA; 4 Information Services, Medical College of Wisconsin, Milwaukee, WI USA

## Abstract

Model organism research is essential for discovering the mechanisms of human diseases by defining biologically meaningful gene to disease relationships. The Rat Genome Database (RGD, (https://rgd.mcw.edu)) is a cross-species knowledgebase and the premier online resource for rat genetic and physiologic data. This rich resource is enhanced by the inclusion and integration of comparative data for human and mouse, as well as other human disease models including chinchilla, dog, bonobo, pig, 13-lined ground squirrel, green monkey, and naked mole-rat. Functional information has been added to records via the assignment of annotations based on sequence similarity to human, rat, and mouse genes. RGD has also imported well-supported cross-species data from external resources. To enable use of these data, RGD has developed a robust infrastructure of standardized ontologies, data formats, and disease- and species-centric portals, complemented with a suite of innovative tools for discovery and analysis. Using examples of single-gene and polygenic human diseases, we illustrate how data from multiple species can help to identify or confirm a gene as involved in a disease and to identify model organisms that can be studied to understand the pathophysiology of a gene or pathway. The ultimate aim of this report is to demonstrate the utility of RGD not only as the core resource for the rat research community but also as a source of bioinformatic tools to support a wider audience, empowering the search for appropriate models for human afflictions.

**Supplementary Information:**

The online version contains supplementary material available at 10.1007/s00335-021-09932-x.

## Introduction

A major challenge for preclinical research is finding, or establishing, a good model for the human disease of interest—one that best recapitulates the phenotypic and genomic profile of that disease in the human system. The incorporation of additional mammalian species will allow researchers to leverage rich datasets across multiple species to find the best model for their needs. Pathogenesis is a complex process that may look different in various species but often involves common pathways, mechanisms, or genetic risk factors. Many animal models are studied to understand human biology and disease. Integrating multidimensional data from multiple species may aid in understanding human disease.

RGD (https://www.rgd.mcw.edu, (Smith et al. 2020)) is recognized as a comprehensive data resource for laboratory rat (*Rattus norvegicus*) as a model for the study of human disease (Laulederkind et al. 2019)*.* Recognizing that the best model system to study human disease depends on the disease etiology and the specific research question, the wide-ranging bioinformatics platform for rat at RGD is enhanced by integration of other mammalian species’ genomic and phenotypic data, particularly human, making it ideally suited for facilitating translational research at a single point of access. Researchers frequently assemble information from more than one model organism when investigating gene–disease relationships. The platforms and tools at RGD support this type of data exploration in a consistent way. RGD has been the front-runner in this endeavor, even before the first public release of the rat reference genome in 2004 (Gibbs et al. 2004).

**Fig. 1:**
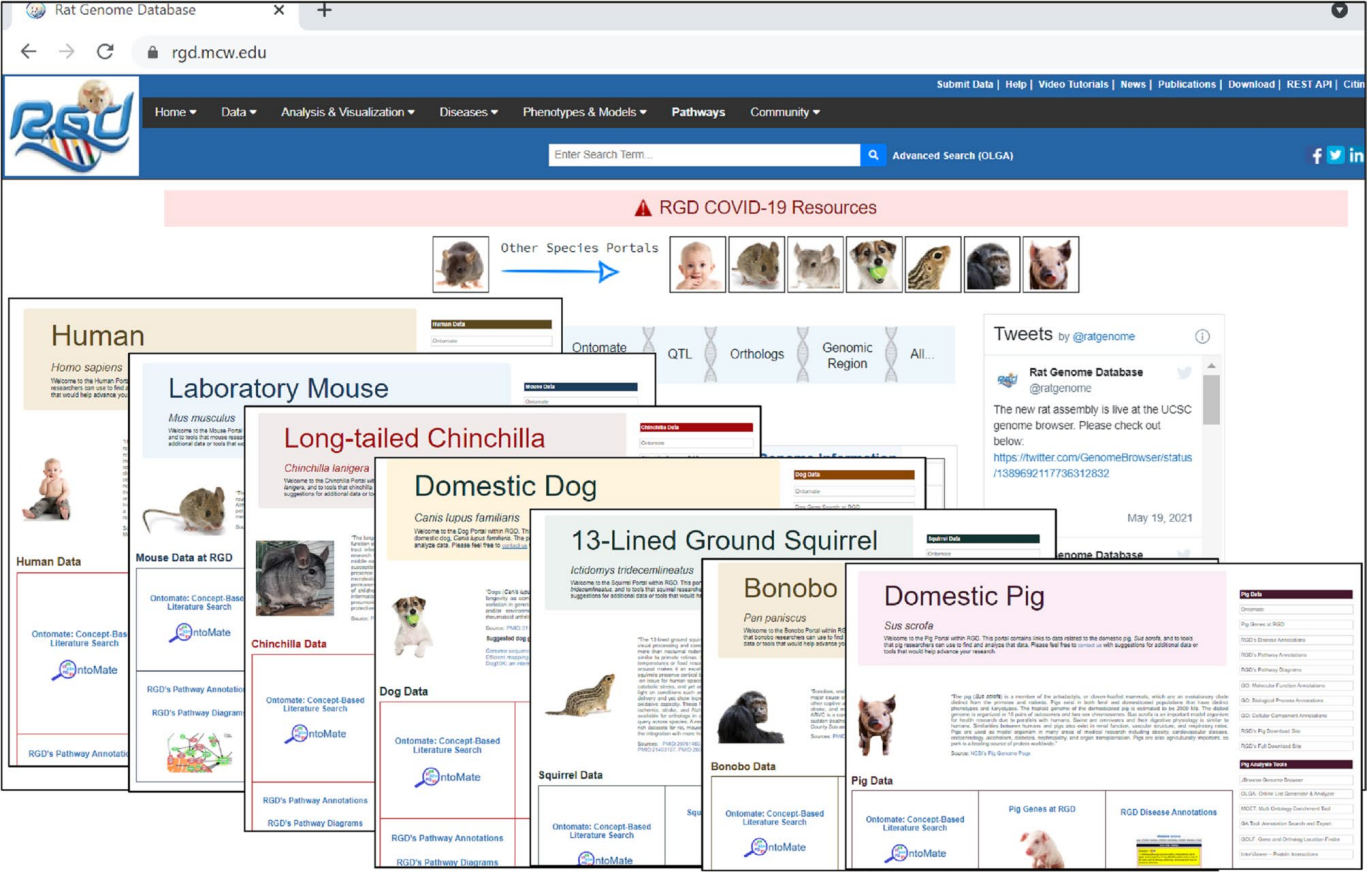
Species-specific landing pages: the RGD homepage offers links to species-centric portals, which provide consolidated access to data and tools

The ability to probe mechanisms of human disease by scrutinizing the available genomic and phenotypic information in rat and mouse has always been a primary objective at RGD. The rat has been studied as a model for human physiology and disease for well over 160 years (Smith et al. 2019). Decades of targeted inbreeding and more recent developments in genome editing in the rat have provided researchers with the ability to produce strains that develop abnormal conditions which more or less mimic human diseases (Aitman et al. 2008; Szpirer 2020, 2021; Chenouard et al. 2021). Combined with a tractable size, social and generally docile behavior, and a relatively short reproductive cycle, the rat is a model of choice for many studies. However, rat is not always the best model, and researchers need to be able to determine and utilize whichever models most closely mimic the human profile for their disease of interest. In addition, rat physiologic and genetic research often incorporate or build upon studies in species such as mouse to best answer research questions. The goal of RGD is to enable exploration of genes that are functionally related in human and model organisms by providing a highly integrated platform that leverages the extensive body of genetic, genomic, and biologic data available. The addition of mammalian species that are models for human disease and the provision of tools designed for researchers further support discovery about health and disease (Fig. 1). Multiple facets of data have been expertly curated and organized by controlled vocabularies for querying and browsing by biomedical researchers. Data involving genes, strains, and QTL annotated to disease, gene ontology (GO), molecular pathway, gene–chemical interactions, and qualitative and quantitative phenotype are available for downloading or manipulating within RGD’s toolbox. Gene–disease relationships are annotated based on published papers, particularly for rat, human, and mouse. Primary annotations are made to genes of whichever species is utilized in the published report. Annotations are then propagated to genes in other species based on orthology and denoted with an “Inferred from Sequence Orthology” (ISO) evidence code. Additional data are imported from well-recognized resources such as the National Center for Biotechnology Information (NCBI; https://www.ncbi.nlm.nih.gov/) (NCBI Resource Coordinators 2018) and its ClinVar database (https://www.ncbi.nlm.nih.gov/clinvar/) (Landrum et al. 2020), Mouse Genome Informatics (MGI; http://www.informatics.jax.org/) (Bult et al. 2019), Ensembl (https://www.ensembl.org/index.html) (Howe et al. 2021), Online Mendelian Inheritance in Man (OMIM; https://www.omim.org/) (Amberger et al. 2015), Online Mendelian Inheritance in Animals (OMIA; https://www.omia.org/home/) (Nicholas 2021), Gene Ontology Consortium (GOC, http://geneontology.org/) (Ashburner et al. 2000; Gene Ontology Consortium 2021), UniProtKB (https://www.uniprot.org/help/uniprotkb) (UniProt Consortium 2019), miRGate (http://mirgate.bioinfo.cnio.es/miRGate/) (Andres-Leon et al. 2015). More recently, RGD expanded to incorporate RNA-Seq expression values with the associated detailed, standardized metadata from GEO (https://www.ncbi.nlm.nih.gov/geo/) (Barrett et al. 2013) and Expression Atlas (https://www.ebi.ac.uk/gxa/home) (Kapushesky et al. 2010) for rat and human, and from GTEx (https://gtexportal.org/home/) (GTEx Consortium 2013) for human. RGD gene pages also link to external gene expression analysis resources such as PhenoGen for rat and mouse (Bhave et al. 2007).

To enable the use of these data, RGD has developed a robust infrastructure with a suite of innovative tools and portals for data discovery and analysis. These include the Object List Generator and Analyzer tool (OLGA), the Gene Annotator (GA) tool for exploring functional annotations for a list of genes, interactive Pathway Portal diagrams, InterViewer for visualizing protein–protein interactions, species-specific JBrowse genome browsers, and RGD's Disease Portals which present consolidated data for disease categories. Variant Visualizer was developed to investigate rat genetic variants but has been expanded to include human variants from ClinVar and variants in numerous sequenced dog breeds (Laulederkind et al. 2019; Smith et al. 2020).

Manual curation is vital to the incorporation and standardization of data in RGD, as it is for all model organism databases. These data are propagated to several external databases such as NCBI and Ensembl and provide key functional information identified in the laboratory rat and other species. Curators at RGD identify relevant literature found in PubMed to assign ontology terms to genome objects using OntoMate, a text-mining tool that automatically tags PubMed abstracts with gene, species, and ontology information (Liu et al. 2015). The ultimate aim of this curation is to enhance the utility of RGD not only for the core community of rat researchers but also to empower researchers with a cross-species knowledgebase to drive discoveries that have the potential to improve human health. This expanded offering of data for multiple species and the analysis tools to easily and efficiently leverage these data, give researchers an excellent resource for discovering precision models for their diseases and/or genes of interest.

## Creating a cross-species platform for human disease model organisms

The study of genomics and disease is greatly benefitted by the inclusion of multiple species for comparative analyses (Prentice and Webster 2004; Howe et al. 2018; Alliance of Genome Resources Consortium 2020; Baldridge et al. 2021). This concept is widely accepted and has led to the development of a series of comparative tools (for example, Harris et al. 2004; Buels et al. 2016; Mungall et al. 2017; Tholey et al. 2017; Dunn et al. 2019; Ruiz-Arenas et al. 2020; Smith et al. 2020; Foley et al. 2021)). From its inception, RGD has championed comparative genomics, initially providing comparative maps and genomic and phenotypic data for rat, mouse, and human (Table 1) (Kwitek et al. 2001; Twigger et al. 2002, 2004) with the goal of better understanding human disease and pathophysiology by integrating data from rat, the major historical physiological and pharmacological model, and mouse, the major genetic model. The importance of centralizing data from multiple species was also recognized with the recent creation of the Alliance of Genome Resources (the Alliance) (Alliance of Genome Resources Consortium 2020), a centralized database with harmonized data across six established databases for model organisms (yeast, *C. elegans, Drosophila*, zebrafish, mouse, rat) and the Gene Ontology Consortium (GOC). RGD is a founding member of the Alliance and a member of the GOC.

**Table 1:** Data incorporated into RGD in 2000 when the database was established

Species	Rat	Human	Mouse
Genes	1987	14	857
Markers (SSLP, EST)	19,562		
Strains	76		
Maps/Assemblies	5	1	

RGD also recognized there is utility in leveraging disease models in species less often considered a traditional model organism and, beginning with chinchilla in 2014, has expanded to include genomic and phenotypic data from eight mammalian species, with the release of two additional species imminent (Table 2), the selection of which has been primarily based on user requests and prioritization of models of human disease with an emphasis on diseases of the heart, lung, and blood. Extensive breed diversification makes *Canis lupus familiaris* (dog) an excellent research model for a wide variety of disorders such as heart disease, cancer, and diabetes (Karlsson and Lindblad-Toh 2008; Davis and Ostrander 2014), and the species has an array of breeds that have been sequenced, which provides comprehensive genomic variation information (Plassais et al. 2019). *Pan paniscus* (bonobo) spontaneously develops a number of human disease conditions including hypertension and cardiomyopathies (Lowenstine et al. 2016; Strong et al. 2016; Celestino-Soper et al. 2018). Although most commonly thought of as agricultural, *Sus scrofa* (pig) is also a model for studies of cardiovascular disease, skin conditions, brain physiology and function, surgical procedures, and the microbiome (Ploug et al. 2008; Samulin et al. 2009; Tsang et al. 2016; Warr et al. 2020). *Ictidomys tridecemlineatus* (13-lined ground squirrel) is an established model for retinal function and dysfunction, and for hypoxia/reperfusion injury due to long spells of hibernation/torpor interspersed with short periods of euthermic arousal (Storey 2010; Luu et al. 2016; Ballinger et al. 2017; Tessier et al. 2019). *Chinchilla lanigera* (long-tailed chinchilla) is often used to study the development, physiology, and pathophysiology of the auditory system (Shimoyama et al. 2016). Most recently, RGD incorporated data of *Chlorocebus sabaeus* (green monkey), which is used in research as a model of neurodegeneration, diabetes, hypertension, and HIV/AIDS (Martin et al. 1990; Palmour et al. 1997). In fact, human immunodeficiency virus (HIV) likely evolved from the simian immunodeficiency virus (SIV), for which the green monkey is the natural host (Compton et al. 2012). *Heterocephalus glaber* (naked mole-rat) is studied for aging/longevity and disease resistance and is also used in social science research on colony structure and communication (Jarvis et al. 1994; Buffenstein 2005).

**Table 2:** Counts of data objects by species

Species	Rat	Human	Mouse	Chinchilla	Bonobo	Dog	13-lined Ground Squirrel	Pig	Green Monkey	Naked Mole rat
Genes	66,544	82,791	75,641	33,266	41,707	52,756	34,418	32,117	41,609	37,447
Proteins	36,395	211,685	88,816	99	43,653	50,115	25,489	122,578	19,525	27,185
Maps/Assemblies	14	20	14	2	4	8	3	4	4	2
Cell Lines	2135	96,476	21,110		33	787	2	248	45	12
Promoters	12,720	66,331	60,623			7,545				
Transcripts	338,715	1,661,329	892,774	100,941	190,658	632,708	89,632	214,434	135,019	74,574
Variants (SNP, Indel)	75,519,796	1,093,201				28,468,158				
Markers (SSLP/EST)	50,134	320,148	55,165							
Strains	4028									
QTL	2378	1911	6828							

Counts reflect data included as of August 2021

Orthologous gene information across species is easily accessed in RGD through a general gene symbol search, using the Gene-Ortholog Location Finder (GOLF) tool, or through RGD gene report pages. Links to orthologs in all species integrated into RGD and additional species at the Alliance can be accessed from each gene report page. In addition, species-centric portals can be accessed directly from the RGD homepage (Fig. 1). These portals give consolidated access to species-specific data and tools, presented in a consistent format for ease of finding and using relevant information. An OntoMate link is provided with the search preset for the species of interest. Collated links to manual and imported disease and pathway annotations, GO annotations, and RGD's data download site are also included in each species portal, along with direct access to all RGD analysis tools, geared toward that species, as well as NCBI and Ensembl species-specific pages. Another important feature of the species portals is a collection of relevant external resources, a curated sampling of selected publications and review articles, and websites considered for informational value on that species.

## Multiple species can model monogenic disease—Wilson disease

Investigators often select a model system that best allows them to address their research question, often that is to investigate a human disease or other biomedical or basic science study. If that disease is monogenic, the availability of cross-species genetic models offers ideal opportunities to study a disease mechanism in the model with the most similar pathophysiology to the human disease. An example of a disease in humans that is modeled by genetic defects in multiple species is Wilson disease (Buiakova et al. 1999; Meng et al. 2004; Merle et al. 2006; Fieten et al. 2012, 2016; Reed et al. 2018; Gerosa et al. 2019; Saba et al. 2019). Wilson disease is a rare autosomal recessive disease characterized by a severe buildup of copper in the brain, liver, and other organs, as a result of abnormal copper metabolism, which results in liver and neurological damage. Although there are a number of other genes that serve as biomarkers and modifiers of the disease, Wilson disease is primarily a monogenic disease caused by mutations in the *ATP7B* gene. Using cross-species data from RGD and the Alliance, and analysis tools available in RGD, a Wilson disease researcher can find extensive evidence supporting the relationship between *ATP7B* and Wilson disease and has the ability to choose the best model organism for further studies of mechanism or potential treatments.

**Fig. 2:**
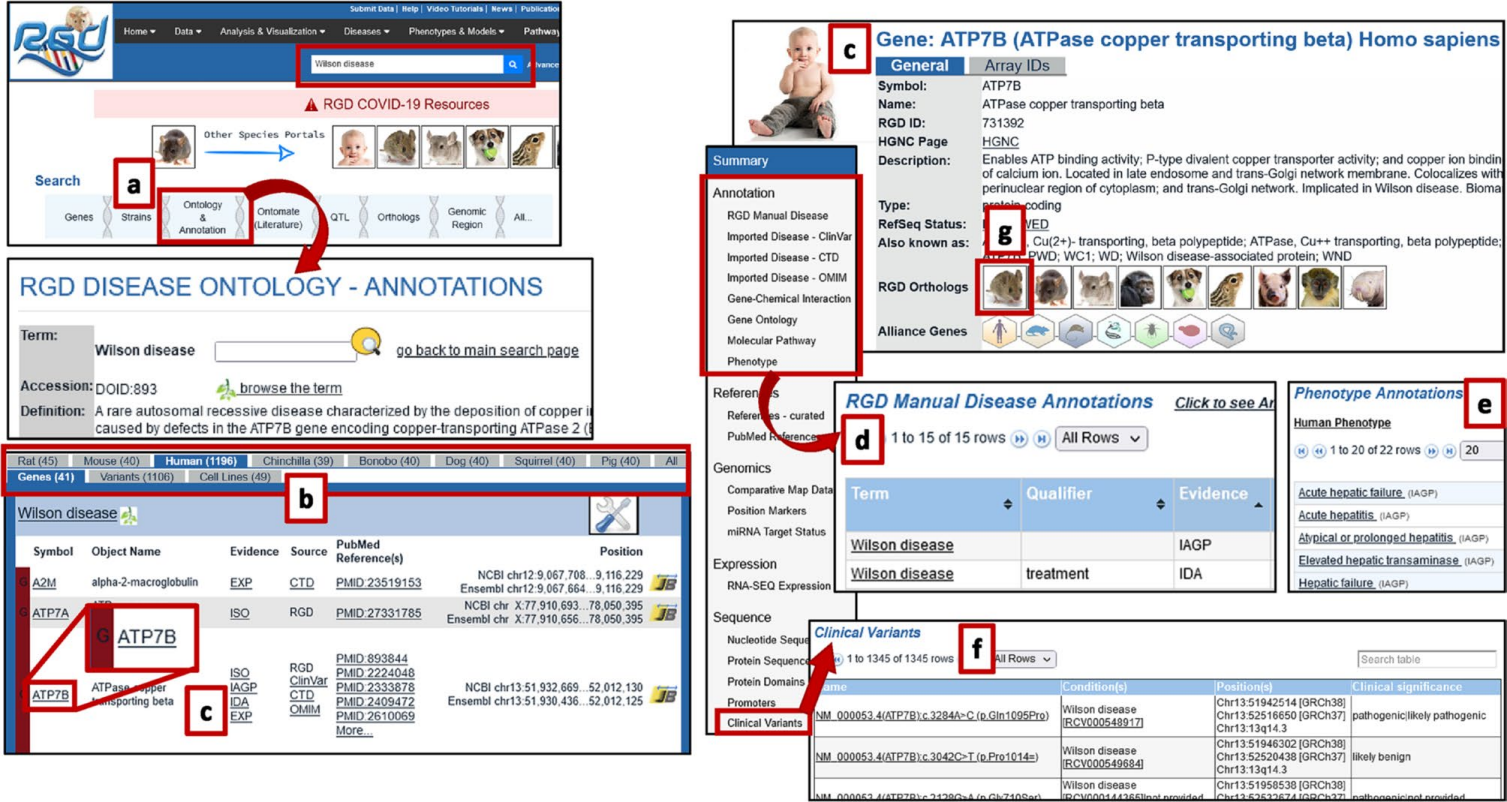
Integrated phenotypic and genotypic data annotations for human Wilson disease: **a** RGD’s general search bar or Ontology & Annotation search finds disease data; **b** ontology report page for Wilson disease contains data for all of RGD’s species; **c** selecting Human in the species list opens Wilson disease annotated genes, and the human *ATP7B* gene symbol links to the gene report page; **d** annotations to Wilson disease found on the gene report page; **e** phenotype annotations; **f** clinical variants; and **g** clickable icons for finding the gene report page for other species such as mouse

Data related to Wilson disease are easily accessed by searching for the term Wilson disease in RGD’s general search or “Ontology & Annotation” search (Fig. 2a). As shown in Fig. 2b, the ontology report page for Wilson disease contains data for all of RGD’s species. On the Wilson disease ontology report page, the result table for human shows data for cell lines, variants, and genes, including *ATP7B*, annotated to the term in the Disease Ontology. A number of these annotations have been manually curated from the literature by RGD or by the Comparative Toxicogenomics Database (CTD), in addition to gene annotations derived from over 1100 ClinVar variants that have been associated with Wilson disease. The human *ATP7B* gene page (Fig. 2c), accessible from the ontology report page by clicking the gene symbol, shows annotations to Wilson disease (Fig. 2d) as well as related diseases such as acute liver failure and liver cirrhosis and phenotypes such as atypical or prolonged hepatitis, high nonceruloplasmin-bound serum copper, polyneuropathy, hepatomegaly, and hepatic failure (Fig. 2e). Both the disease annotations imported from ClinVar and the Clinical Variants section of the human gene page (Fig. 2f) give links to RGD records for individual *ATP7B* variants associated with Wilson disease.

**Fig. 3:**
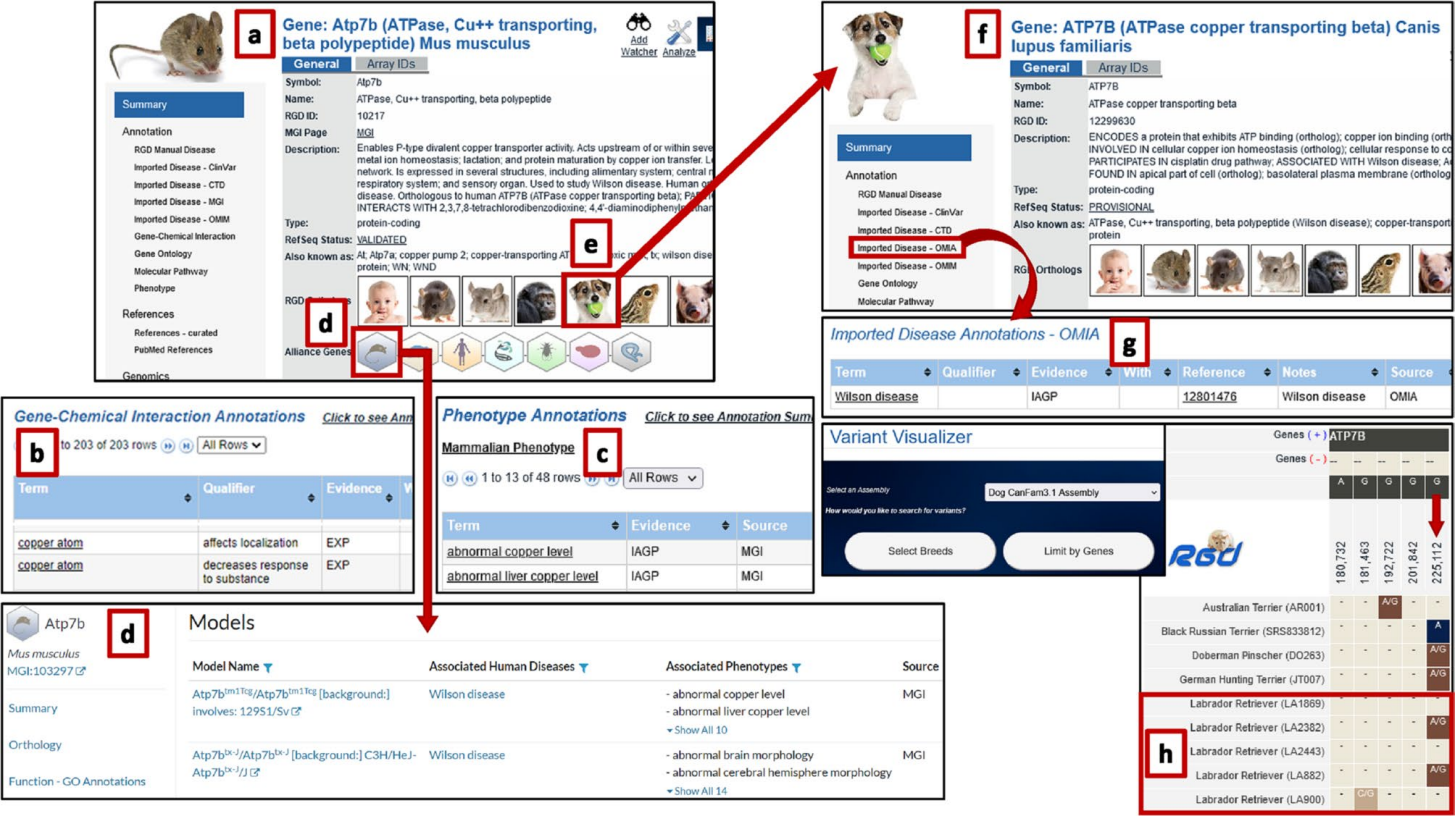
Multispecies data identify multiple *Atp7b* models for Wilson disease: **a** the mouse gene report page provides access to annotations such as **b** ChEBI gene–chemical interactions, **c** phenotype annotations; **d** external links to Alliance of Genome Resources website; **e** multispecies links such as the dog icon that will open the **f** gene report page for dog, which shows **g** manually curated annotations imported from OMIA; and **h** RGD’s Variant Visualizer tool with data for an extensive list of breeds, some of which have possibly damaging variants and have been shown to develop Wilson disease

In addition to supporting evidence in human for the association between *ATP7B* and Wilson disease, RGD provides data for several animal models of Wilson disease. The mouse icon in the “RGD Orthologs” section of the human gene page (Fig. 2g) gives one click access to RGD’s mouse gene report page (Fig. 3a). In addition to annotations to Wilson disease, experimental ChEBI annotations imported from CTD (Fig. 3b) give evidence for interactions between copper and the mouse Atp7b protein. Mammalian Phenotype ontology annotations (Fig. 3c) imported from Mouse Genome Informatics (MGI) list numerous phenotypes observed in genetically modified (e.g., knockout) mice related to abnormal copper levels in multiple organs, liver cirrhosis, neurological abnormalities, and postnatal lethality. Applicable models in mouse can be explored via the link to MGI in the “External Database Links” section of the mouse gene report page, or by the mouse icon in the “Alliance Genes” list at the top of the RGD gene report page to navigate to the Alliance of Genome Resources website (Fig. 3d) where additional information is available on mouse models of the disease.

From RGD’s mouse gene page, the dog icon (Fig. 3e) in the RGD orthologs list gives easy access to the dog gene report page for *ATP7B* (Fig. 3f). Labrador Retrievers, Doberman Pinschers, and several terrier breeds have been shown to occasionally develop Wilson disease (Kruitwagen and Penning 2019). RGD's dog *ATP7B* gene page (https://rgd.mcw.edu/rgdweb/report/gene/main.html?id=12299630#importedAnnotationsOMIA) shows this association via a manually curated annotation imported from the Online Mendelian Inheritance in Animals (OMIA) database (Fig. 3g). According to OMIA, Fieten et al. found a G > A mutation at position 225,112 on chromosome 22 in a Labrador Retriever which displayed increased copper accumulation and copper toxicosis (Fieten et al. 2016). In RGD’s Variant Visualizer tool for dog (https://rgd.mcw.edu/rgdweb/front/config.html?mapKey=631), selection of the Labrador Retriever breed group, searching for the *ATP7B* gene, and limiting results to non-synonymous variants returns a heterozygous G > A variant at this position in two of the five samples (Fig. 3h). Clicking on the variant provides access to more detailed information about it, including the information that this variant is predicted to be “possibly damaging” by the Polyphen algorithm. By editing the breed search to include Doberman Pinschers and all available terrier breeds, additional cases of the G > A mutation were seen in a Doberman Pinscher, a Black Russian Terrier, and a German Hunting Terrier. Additional non-synonymous variants were identified in other terrier breeds, including variants having predicted effects ranging from benign to probably damaging.

**Fig. 4:**
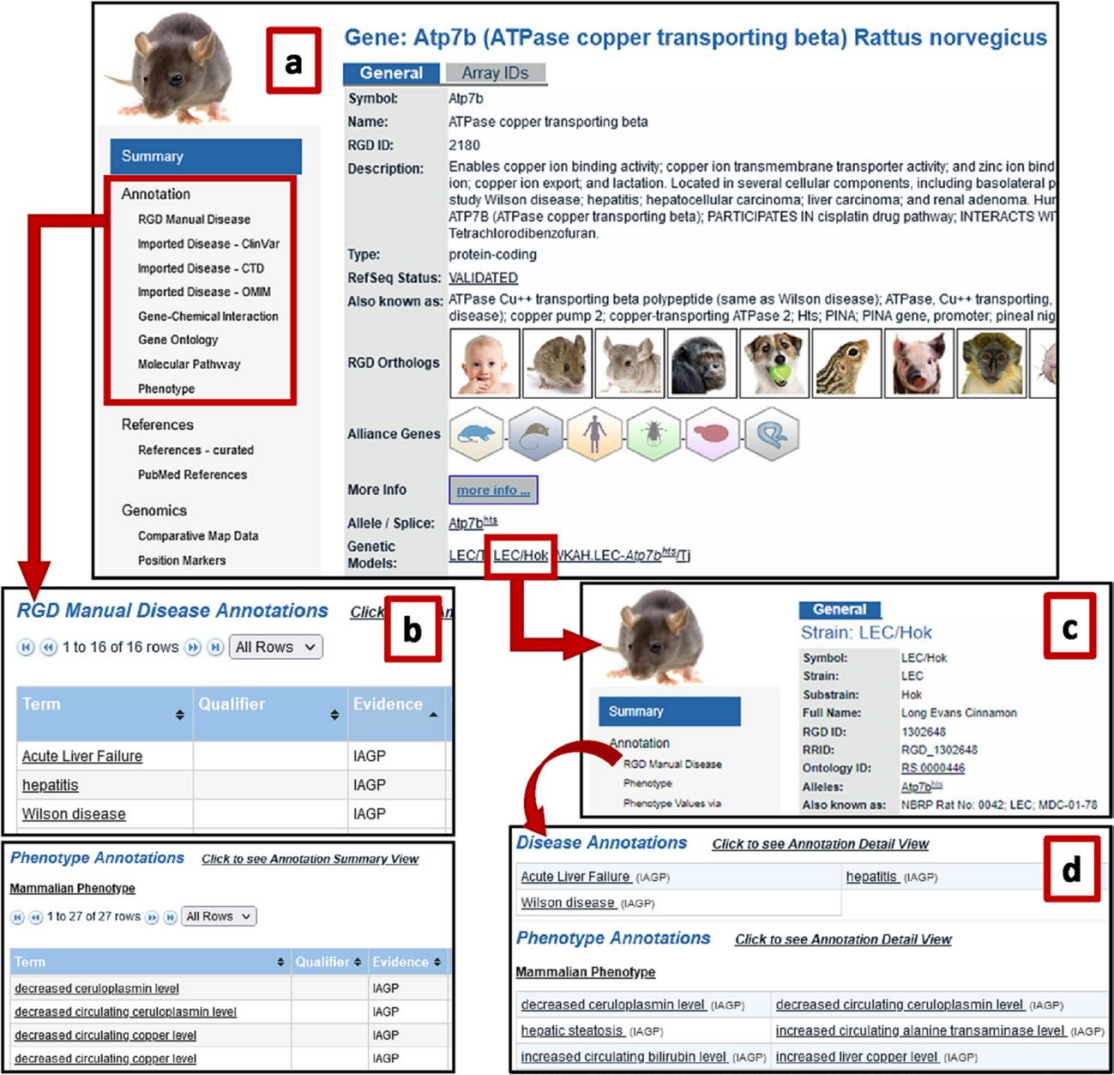
A spontaneous mutation in the LEC/Hok rat makes the strain a model for Wilson disease: **a** the gene report page for *Atp7b* in rat; **b** manual disease annotations for the gene; c. phenotype annotations for the gene; **c** genetic rat model with a mutation in *Atp7b*; and its **d** disease and phenotype annotations

The rat *Atp7b* gene report page (Fig. 4a) has Wilson disease annotations based on phenotypes resulting from a spontaneous mutation in *Atp7b* in a rat with Wilson disease features (Fig. 4b). In addition to annotations to Wilson disease and related Mammalian Phenotype annotations, the rat gene page provides links to the mutant allele and mutant strains with aberrant *Atp7b* genes (Fig. 4c).

These same mutant strains can also be accessed by selecting the Find Models tool (https://rgd.mcw.edu/rgdweb/models/findModels.html) from the “Phenotypes & Models” menu in the header of most RGD pages. Searching for Wilson disease in the tool shows that LEC/Hok is an established model for this condition (Fig. 4c). In the result list, click the strain symbol to access the RGD report page for this strain which details the clinical manifestations of Wilson disease displayed in this mutant rat. When compared to the related LEA/Hok strain, LEC/Hok rats show acute liver failure, hepatitis, decreased circulating ceruloplasmin, increased liver copper, hepatic steatosis, oxidative stress, and marks of liver damage such as jaundice and increased circulating alanine transaminase, aspartate transaminase, and bilirubin levels (Fig. 4d), all of which recapitulate phenotypes seen in Wilson disease patients. Information on the LEC/Hok page lists *Atp7b* as the mutated gene in this strain. A listing of rat models for liver diseases is also accessible through RGD’s Liver Disease Portal page. The portal provides a link to a Rat Strain Models page where rat strains which have been curated as models of liver disease are compiled for ease of access. Similar curated information for rat strain models is provided as a link at the bottom of each of RGD’s Disease Portal pages, updated to reflect the applicable disease category.

## Cross-species studies of complex disease—thrombosis and blood coagulation

**Fig. 5:**
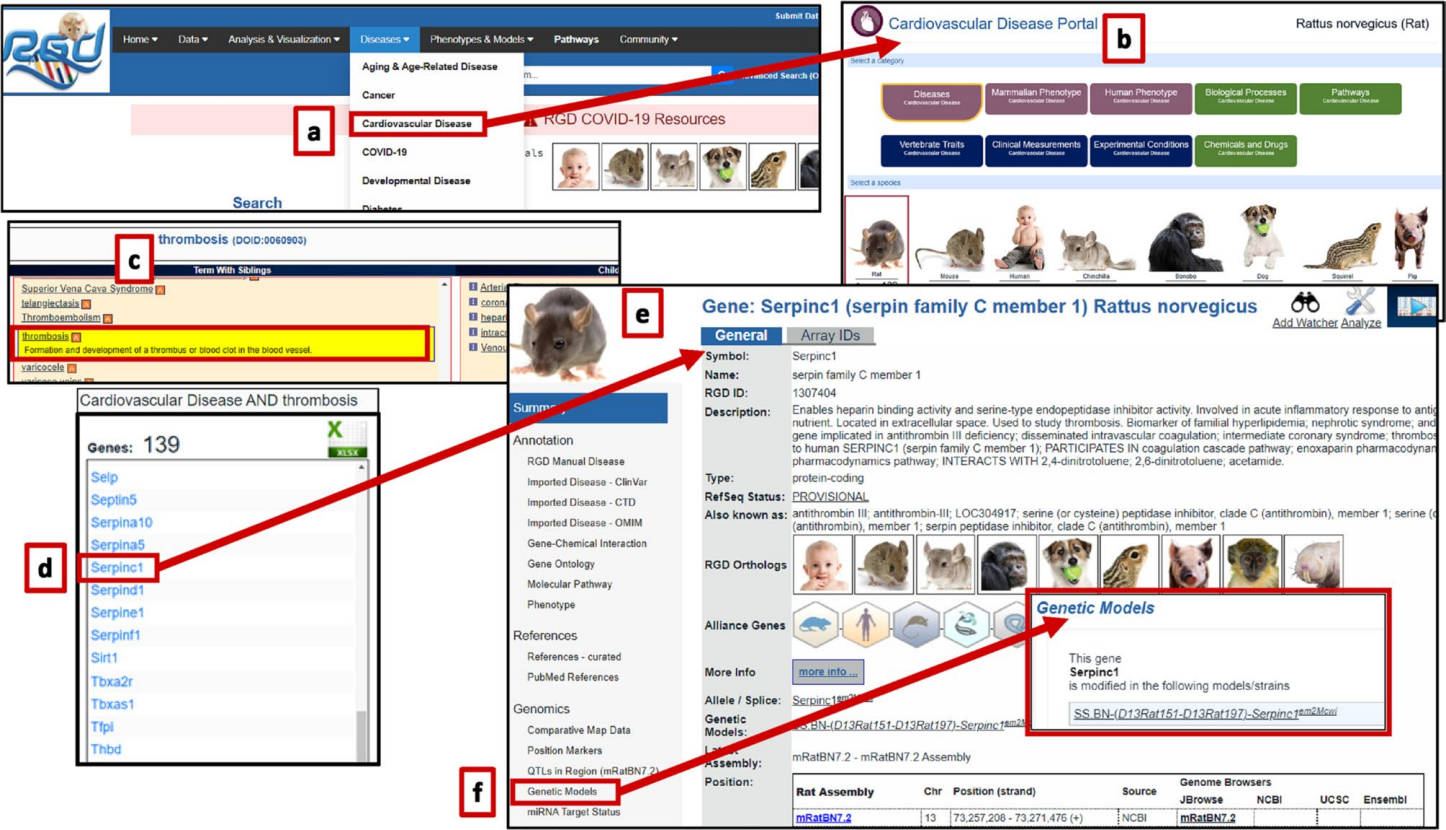
Cardiovascular Disease Portal page for data linking *Serpinc1* to thrombosis in multiple species: **a** Main menu Diseases and **b** the subsequent Cardiovascular Disease Portal; **c** disease ontology is used to narrow the disease term to thrombosis; **d** a more focused disease term results in a shorter list of 139 genes in rat, and *Serpinc1* is selected; **e** the gene symbol is a link that opens the report page for that gene in the selected species; **f** the rat gene report page also provides information for Genetic Models, including in this case SS.BN-(D13Rat151-D13Rat197)-*Serpinc1*^em2Mcwi^ (RGD:12,790,721). For more detailed steps, see Online Resource 1

In contrast to the relatively small number of monogenic diseases, most diseases are complex, involving the action and interaction of environmental stressors and multiple genetic variations with varying effect sizes. In such cases, the use of genomic integration across multiple species can lead to better understanding of disease mechanisms and pathophysiology. As an example, a current topic of intense research interest is clotting mechanisms. While the mechanisms involved in blood clotting have been well established (Farris 1954), recent observations of coagulopathy in the context of viral infections such as SARS-CoV-2 have stimulated a wave of new investigations, e.g., (Abu-Farha et al. 2020; Campbell et al. 2021), particularly in the area of thrombosis. Consolidated data for thrombosis can be found in the RGD Disease Portals (Fig. 5a) (Smith et al. 2020), from the main menu dropdown list. Specifically, selecting the Cardiovascular Disease Portal link (Fig. 5b) provides an integrated ontology browser to facilitate narrowing the data category being displayed (Fig. 5c). In the default view, the term cardiovascular system disease is highlighted in the center panel. Navigate to the child term vascular disease by selecting it in the right panel of the ontology browser. Once selected, it moves to the middle panel and the child term thrombosis can be chosen. While the broad category of cardiovascular system disease has thousands of associated genes, the more specific term of thrombosis gives a more focused list of 139 genes in rat. Annotations for other species can be selected directly by clicking on the species icon. Scrolling down the disease portal page provides the gene list and a genome view of the locations of the associated genes, QTL, and mapped strains, where available. Selecting the “A” icon next to a disease term will link out to a Disease Ontology report page similar to the one described above with annotations for that disease and ontologically related diseases, and with tabs for each species and each associated data type.

From the list of 139 rat genes in the Cardiovascular Disease Portal annotated to thrombosis, a gene of interest can be selected. Here, we have selected *Serpinc1* (Fig. 5d). The gene symbol is a link that will open the report page for the gene in the species selected on the Disease Portal (Fig. 5e). The rat gene report page for *Serpinc1*, for instance, shows RGD manual disease annotations including an experimental annotation to thrombosis. Looking across species, the mouse gene shows an experimental annotation to the more specific child term venous thrombosis. The human gene report page (Online Resource 1) also shows manual, experimental annotations for thrombosis, venous thrombosis, and venous thromboembolism assigned by RGD curators and imported from CTD, as well as annotations based on ClinVar variants. These experimental annotations are then assigned to the orthologous genes in other species as an indicator that, although it has not been shown in those species, the *Serpinc1* genes in those species might also be associated with thrombosis. This inference is indicated by the evidence code "ISO" (Inferred from Sequence Orthology).

Returning to the rat gene page, the Genetic Models section indicates that *Serpinc1* has been mutated in the strain SS.BN-(*D13Rat151-D13Rat197*)-*Serpinc1*^*em2Mcwi*^ (RGD:12,790,721) (Fig. 5f). Although not annotated to thrombosis, this strain has been studied for a role in the severity of renal ischemia/reperfusion injury. Gene Ontology annotations show that rat *Serpinc1* is involved in blood coagulation and has molecular functions that include heparin binding. Reference IDs are given for each annotation, allowing the researcher to pursue the specifics of the connection (Ikezoe et al. 2013; Nakahara et al. 2013).

**Fig. 6:**
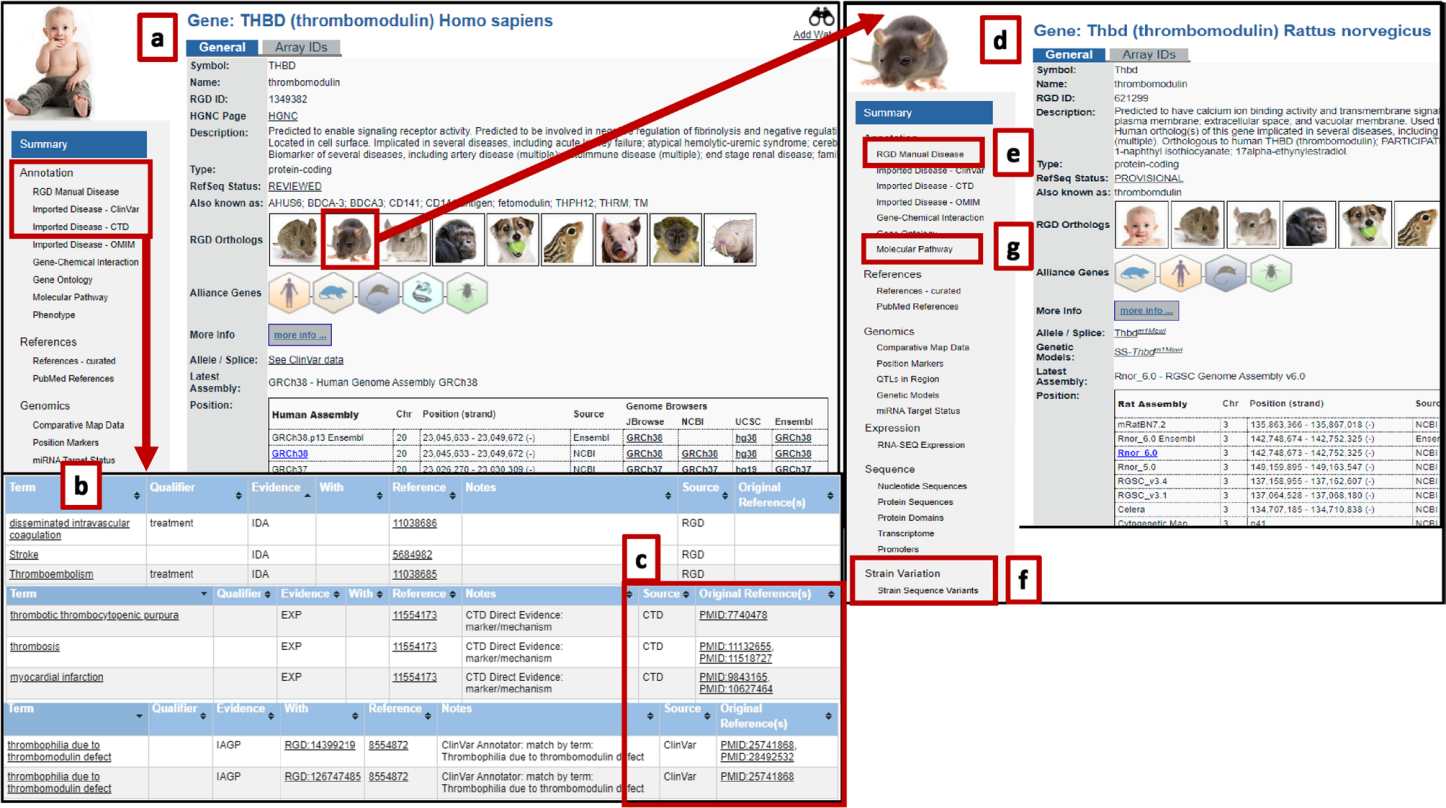
Cardiovascular disease portal page used to find data linking *THBD* to thrombosis in multiple species: **a** The human thrombomodulin (*THBD*) gene page shows **b** manually curated and imported annotations; **c** links to the original publications/originating databases; **d** selecting the rat species icon will open the gene page for rat with; **e** manual disease annotations; **f** gene variations in rat strains; and **g** molecular pathway annotations and links to the Pathway Ontology. For more detailed steps, see Online Resource 2

Choosing a different gene from the disease portal page, thrombomodulin (*Thbd*), will take the user to that specific gene report page (Fig. 6 and Online Resource 2). On the human thrombomodulin gene page, (Fig. 6a) manually curated and imported annotations can be found for references indicating that human recombinant thromobomodulin was used as a treatment for coagulopathy in mice and indicating its use in disseminated intravascular coagulation in humans (Fig. 6b) (Ikezoe et al. 2013; Nakahara et al. 2013). There is also evidence that *THBD* could be involved in related disease conditions in humans, notably stroke, myocardial infarction, and other thromboembolytic conditions (e.g., Kunz et al. 2000; Pilarska et al. 2010). Drilling into the information available on the gene page for human, RGD manual disease annotations are available that serve as a distillation of the salient information from the research while providing the user easy access to supporting research publications. Likewise, annotations from ClinVar, CTD, OMIM, and the Human Phenotype Ontology group provide both focused data and links to the original publications (Fig. 6c) and/or the originating databases for additional information. Clinical variants imported from ClinVar are listed on the gene page, complete with associated conditions, chromosomal locations, and potential significance. In rat (Fig. 6d), this gene has been manually annotated for acute injury/failure for kidney, liver, and lung, particularly related to ischemia, but to coagulopathy only through gene orthology (Fig. 6e). There are genomic variants in a number of rat strains (Fig. 6f), also listed on the gene report page and available in Variant Visualizer, and a *Thbd*-mutant rat strain (SS-Thbd^m1Mcwi^, RGD:1,642,273) has been generated.

**Fig. 7:**
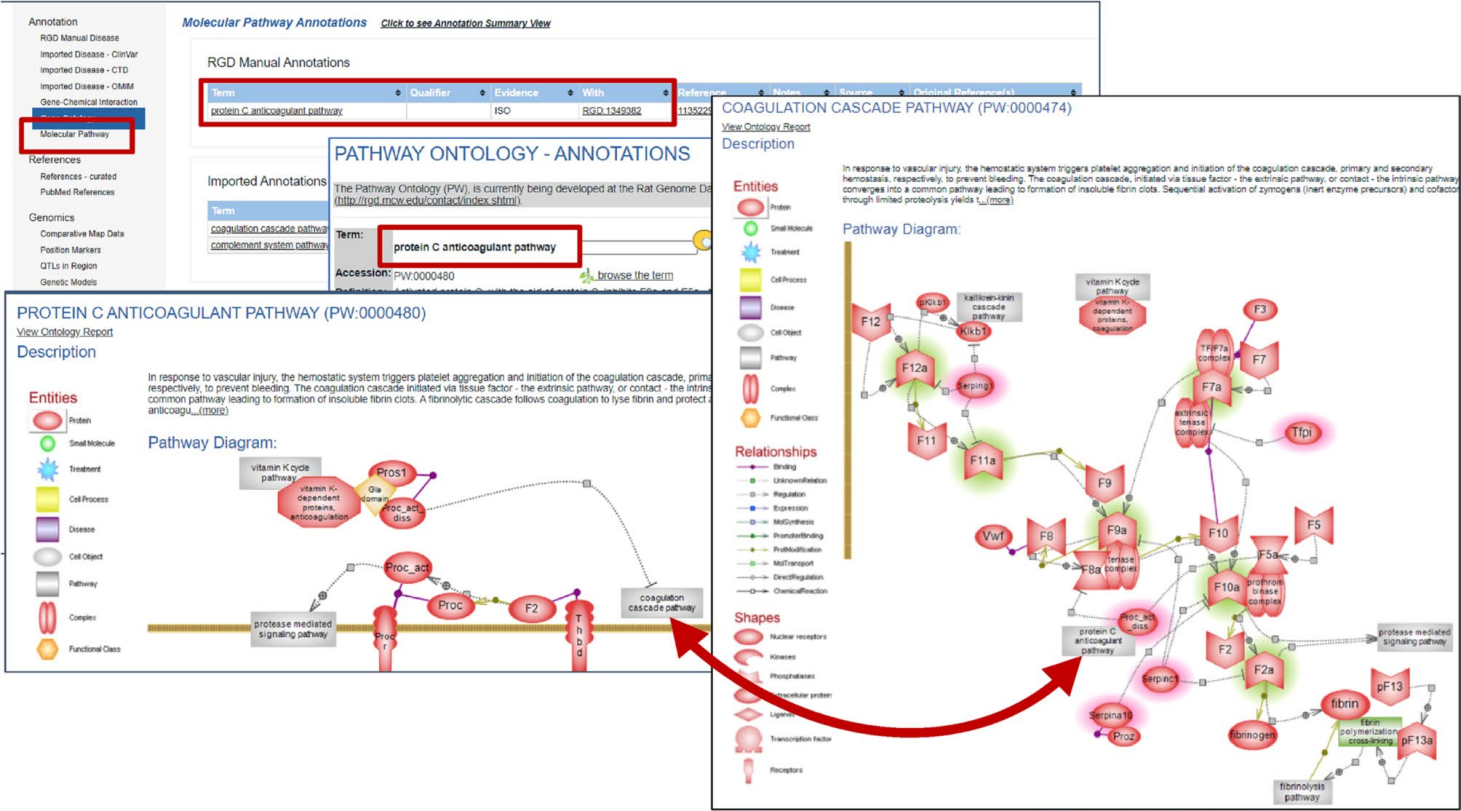
Molecular pathway annotations and interactive pathway diagrams for *Serpinc1* and *Thbd*: Molecular Pathway Annotations are clickable links that open the Pathway Ontology—Annotations page, from which one can open the protein C anticoagulant pathway and the coagulation cascade pathway diagram. The two pathways are bidirectionally linked

Selecting Molecular Pathway (Fig. 6g) in the left sidebar of the *Thbd* gene report page and following the annotation to protein C anticoagulant pathway links to the term’s ontology report page showing the list of six genes involved in the pathway, including *Thbd*. At the top of this page is a link to RGD's interactive pathway diagram for the protein C anticoagulation pathway (Fig. 7). This diagram in turn bidirectionally links to the coagulation cascade pathway diagram. The coagulation cascade is the series of events proceeding via the tissue factor (extrinsic) or the contact activation (intrinsic) pathway and converging in the formation of fibrin clots. Both *Thbd* and *Serpinc1* participate in the anticoagulation systems that are in place to modulate the pathway which, when functioning correctly, prevent excessive clotting. The coagulation cascade and the complement pathway of immune responses are connected in a cross-talk. These diagrams are based on terms in the Pathway Ontology at RGD and offer visualization of molecular pathways and the interacting partners.

## From genes to mechanisms or treatments

**Fig. 8:**
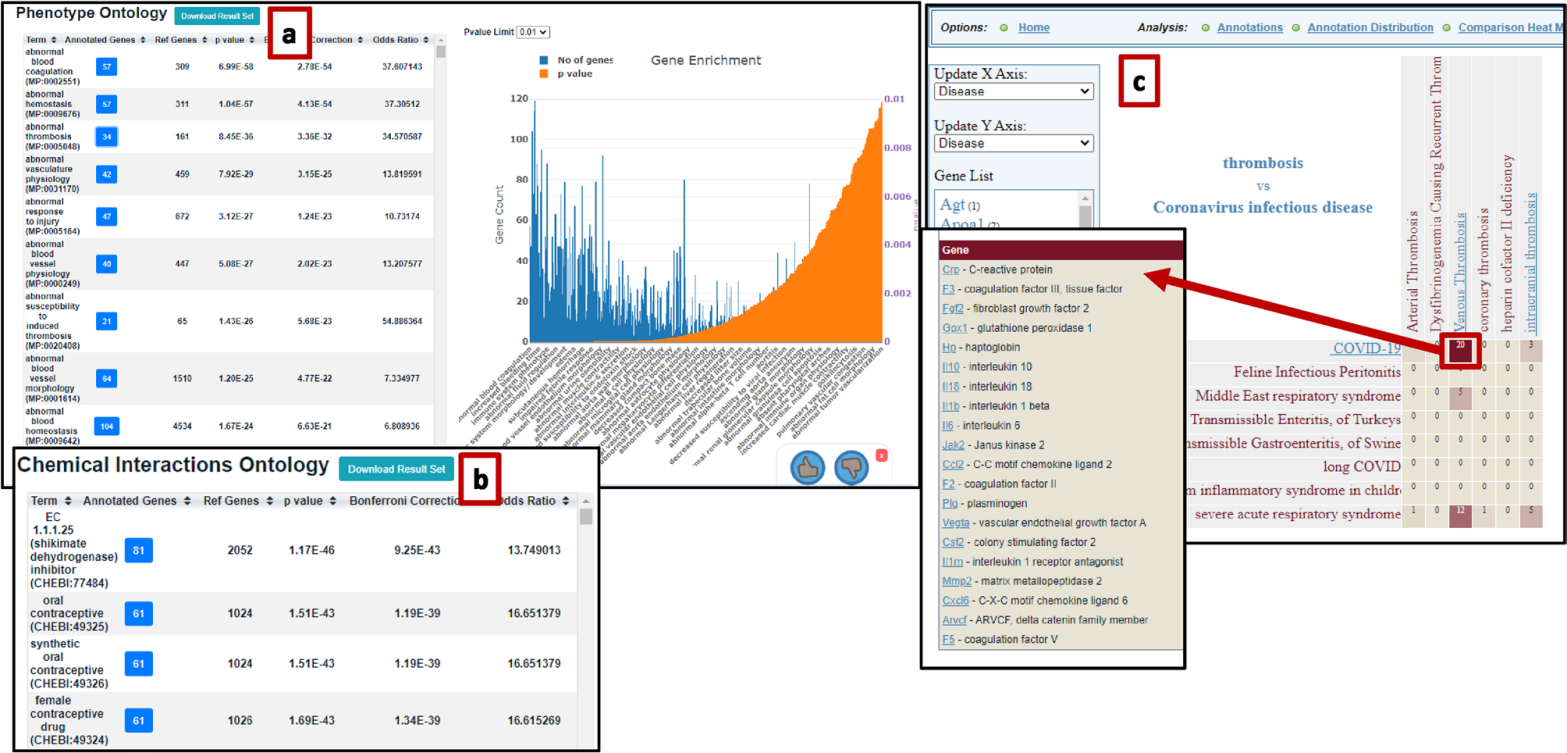
Using functional analysis tools from the RGD toolkit to interrogate the thrombosis gene list: Within the Cardiovascular Disease Portal, with the specific disease selection for thrombosis, one finds 139 genes annotated in rat, as detailed in Fig. 5 and Online Resource 1. **a** Selecting “mouse” and choosing “MP: Phenotype Ontology” enrichment launches an analysis that utilizes MOET; **b** selecting “rat” and “CHEBI: Chemical/Drug Enrichment” alters the results accordingly; **c** downloading the gene list into Excel and entering it into the Gene Annotator (GA) Tool will return all annotations in any ontology selected and provide a feature of the GA tool that can generate a comparative heatmap of the genes in the intersections between and within ontologies. Here, we show the resultant heatmap of genes for the intersection of disease ontology child terms for thrombosis and Coronavirus infectious disease. For more detailed steps, see Online Resource 3

One of the most powerful RGD functions is the ability to utilize analysis tools to interrogate a gene list leveraging our integrated data infrastructure. One such tool is the Multi-Ontology Enrichment Tool (MOET). MOET can analyze enrichment of a species-specific gene list annotated to a selected term, not just individual genes in the list. Within the Cardiovascular Disease Portal, with the specific disease selection for thrombosis, one finds 139 genes annotated in rat and 140 in mouse, as detailed in Fig. 5. Selecting “mouse” and choosing “MP: Phenotype Ontology” enrichment launches an analysis that utilizes MOET (Fig. 8a and Online Resource 3). The most highly enriched terms for this list of genes are closely related to thrombosis, hemostasis, blood coagulation, and vascular physiology. As detailed in Online Resource 3, further analysis is possible by navigating the initial results page. Selecting any one phenotype category within the enrichment list will display the list of genes annotated to that term. One can choose “Explore This Gene Set,” which will open the full MOET tool and provide additional options. Alternatively, a gene from the dropdown list can be selected to open the gene report page. Within the MOET tool, one may select a different species, a different ontology, or a subset list. For example, selecting “rat” and “CHEBI: Chemical/Drug Enrichment” (Fig. 8b), three of the top four overrepresented terms for the list of thrombosis-related genes are contraceptive drug terms; interestingly, there have been studies showing that use of contraceptive drugs can be associated with DVT and pulmonary embolism (Sitruk-Ware 2016). Selecting the term for oral contraceptives will open the gene list (see Online Resource 3).

Using the Excel download function, one can capture the thrombosis gene list from the Cardiovascular Disease Portal. This list can serve as input for a number of tools within the RGD toolbox (Online Resource 3). It is then possible to employ the power of multiple species and multiple ontologies to interrogate genetic associations, protein alterations, and interactions, even evaluate therapeutic targets. The Gene Annotator (GA) Tool, accessible from either the homepage icon or from the Analysis and Visualization dropdown menu, can accept a gene list for any species and find disease, phenotype, pathway, GO, and ChEBI annotations for all of the genes on the list across all of RGD’s species. One can select individual ontologies, gene information sources, and species, or select all (Online Resource 3). The analysis results return all annotations in any ontology selected. A feature of the GA tool is the ability to generate a comparative heatmap of the genes in the intersections between and within ontologies. Here, we show the resultant heatmap of genes for the intersection of disease ontology child terms for thrombosis and Coronavirus infectious disease (Fig. 8c). The cell at the intersection of the Venous Thrombosis column and the COVID-19 row is clickable and opens the list of the 20 genes from the original list of 139 which are annotated to both terms (Fig. 8c). Further analysis, detailed in Online Resource 3, is possible within the results page.

## Conclusion

The laboratory rat has been used as a model to study human disease for over 160 years. In many cases, it is the model of choice for such studies, but it is not the best model in every case. For instance, although rat can be used in studies of middle ear infections, chinchilla is the preferred model for such studies because of the striking similarities between the anatomy of the chinchilla ear and that of human children. Likewise, the retina of a 13-lined ground squirrel is far more similar to the primate retina than nocturnal rodents such as rats, making squirrel the preferred model for studies of retinal function. Because of this and because researchers will often use multiple models to obtain as complete an answer as possible to their research questions, resources such as RGD and the Alliance, which integrate data for multiple species, have become even more valuable.

RGD has always had a commitment to providing data across species. This prompted the development of automated pipelines to import data. Because of this, RGD has been able to expand its repertoire of species that are well-studied models for one or more human diseases. The species have been selected and continue to be added on the basis of their status as established models for diseases of interest to RGD users. In addition, for species such as chinchilla and squirrel that are just moving into the realm of genomics and for which there are less species-specific data available, integration with more highly studied species can help to inform research going forward. For less well-studied species, annotations are assigned based on orthology between genes in those species and genes in other species. While there are caveats to this approach (i.e., that gene function can differ in different species), having this information is a significant advantage for less-studied species and/or species that do not have curated databases of their own. Assignment of disease, pathway, and GO annotations based on orthology benefits researchers by leveraging that information to reveal genes for study in their model organism. A squirrel researcher, for example, could go to an RGD disease portal, find gene lists for squirrel that have been associated with disease in other species, and design experiments to determine if there are similar associations in squirrel.

The integration of both imported data from other sources and RGD’s ongoing literature curation, the addition of new rat, bonobo, and other species’ assemblies, and the ability to explore human clinical variants as well as variants in rat strains and dog breeds, all, help to elucidate the genomics of disease across species. The addition of new analysis tools and recent improvements to existing tools as well as links that allow users to directly submit the results obtained with one tool as input for another tool facilitate navigation between tools, simplifying researchers’ analysis workflows and helping users assess the most efficacious next step in their analysis. This manuscript has demonstrated the utility of the integration of multiple species’ genomics into the Rat Genome Database.

## Supplementary Information

Below is the link to the electronic supplementary material.

Supplementary file1 (PDF 1947 KB)

Supplementary file2 (PDF 1018 KB)

Supplementary file3 (PDF 1584 KB)
