# Supplementary material for: The Rat Genome Database (RGD) facilitates genomic and phenotypic data integration across multiple species for biomedical research
Source: Mamm Genome. 2021 Nov 5;33(1):66–80. doi: 10.1007/s00335-021-09932-x (PMC8570235; doi:10.1007/s00335-021-09932-x)
Supplement: Supplementary file 1 — Supplementary file1 (PDF 1947 KB) [file 335_2021_9932_MOESM1_ESM.pdf]

Online Resource (OR)1 supplement figure: Cardiovascular Disease Portal page used to find data linking *Serpinc1* to thrombosis in multiple species.

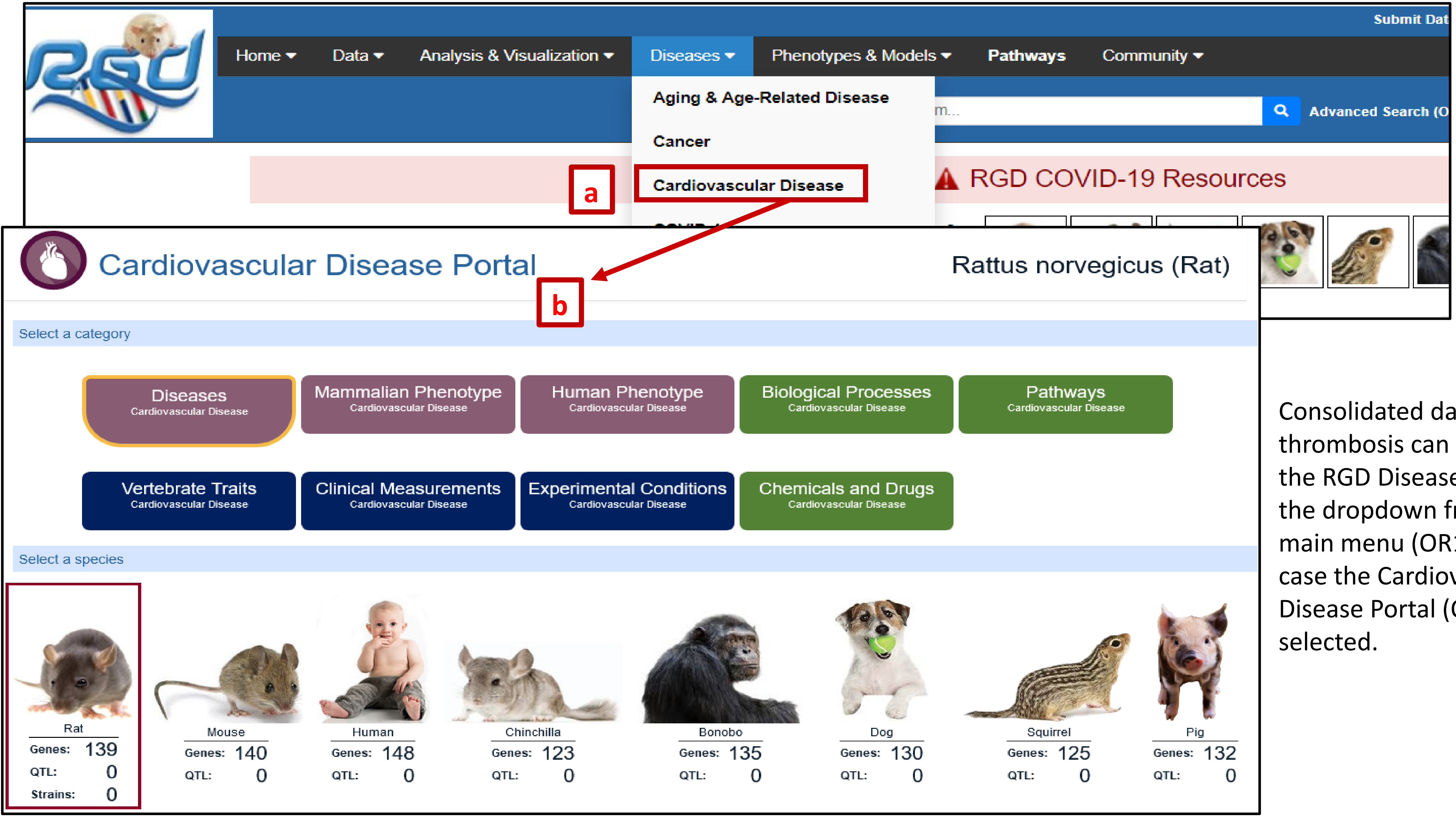

Consolidated data for thrombosis can be found in the RGD Disease Portals, in the dropdown from the main menu (OR1a). In this case the Cardiovascular Disease Portal (OR1b) is selected.

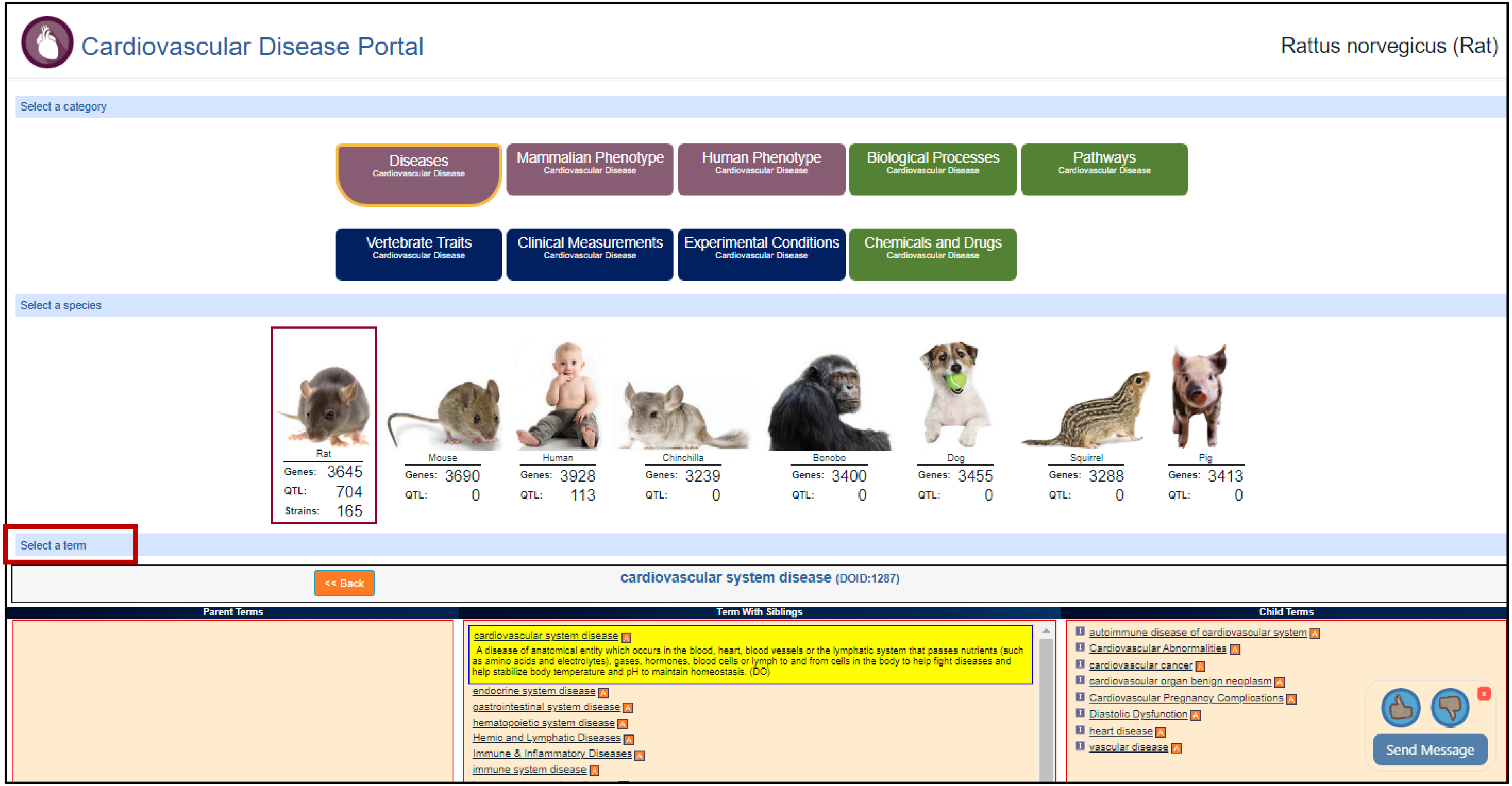

The Disease Portals contain an integrated ontology browser to facilitate narrowing the data category being displayed. Use the Select a term section to walk through the disease ontology terms.

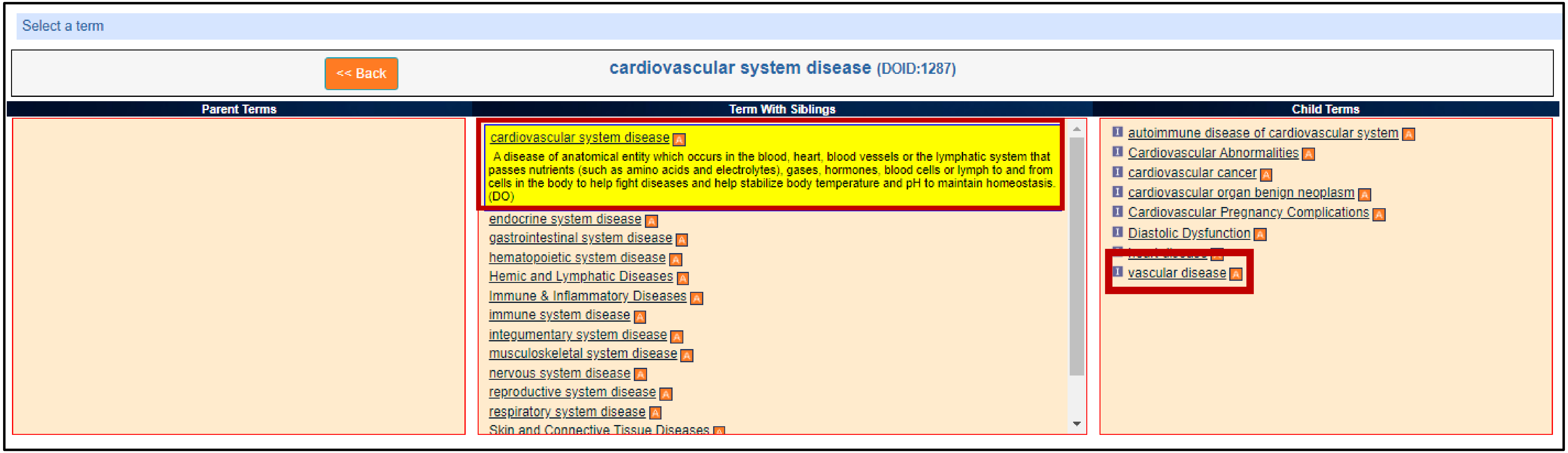

In the default view, the term cardiovascular system disease is highlighted in the center panel. Navigate to the child term vascular disease by selecting it in the right panel of the ontology browser.

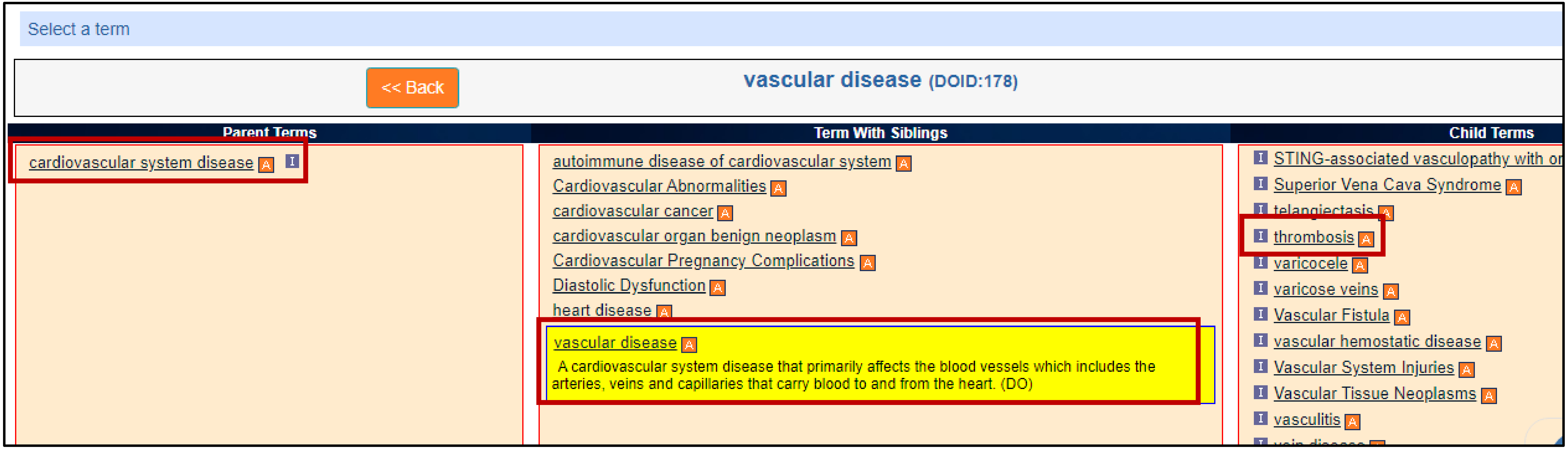

Once selected it moves to the middle pane and the child term thrombosis can be chosen.

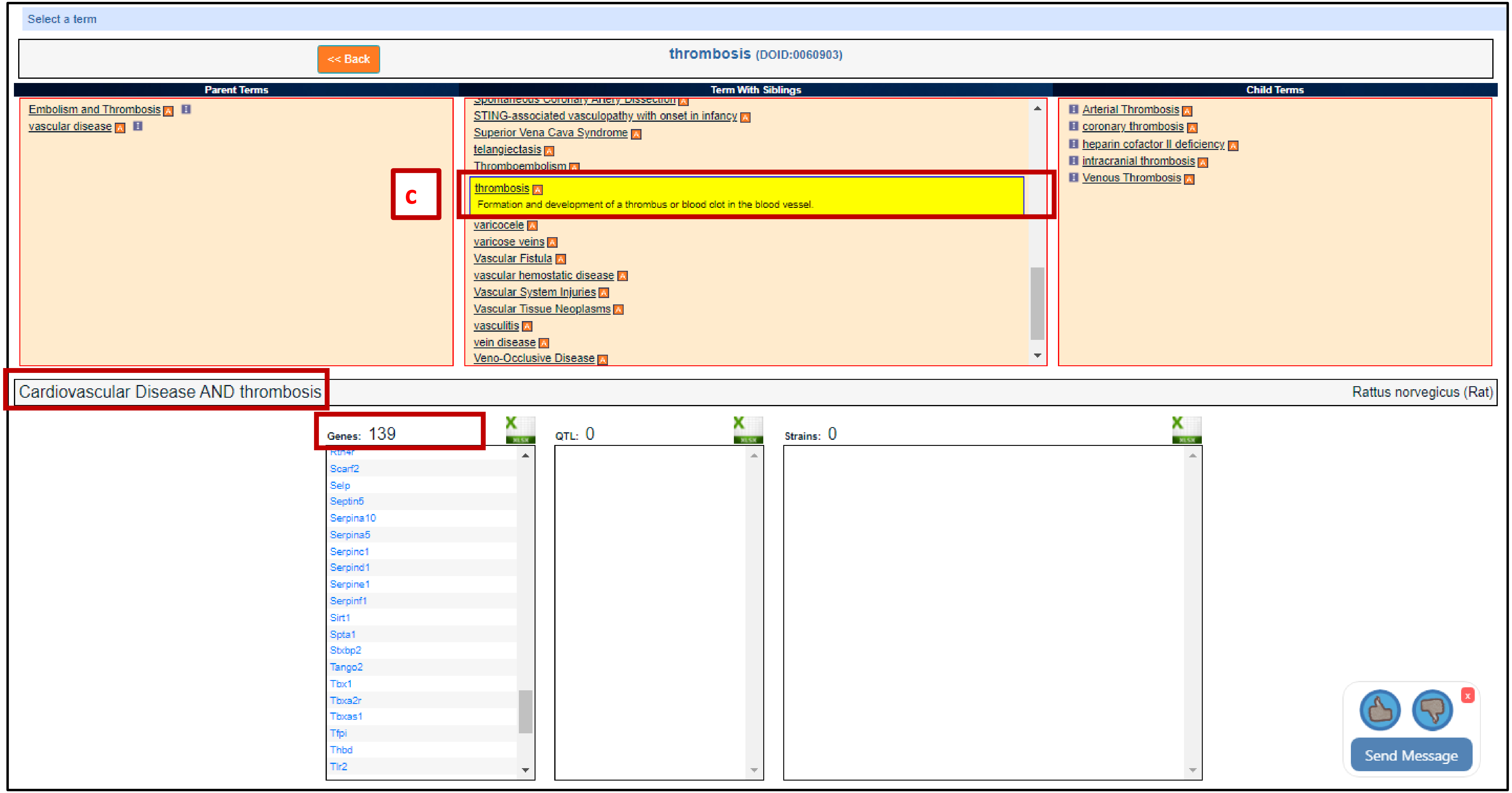

While the broad category of cardiovascular system disease has thousands of associated genes, the selection of the more specific term thrombosis gives a more focused list of 139 genes in rat (OR1c).

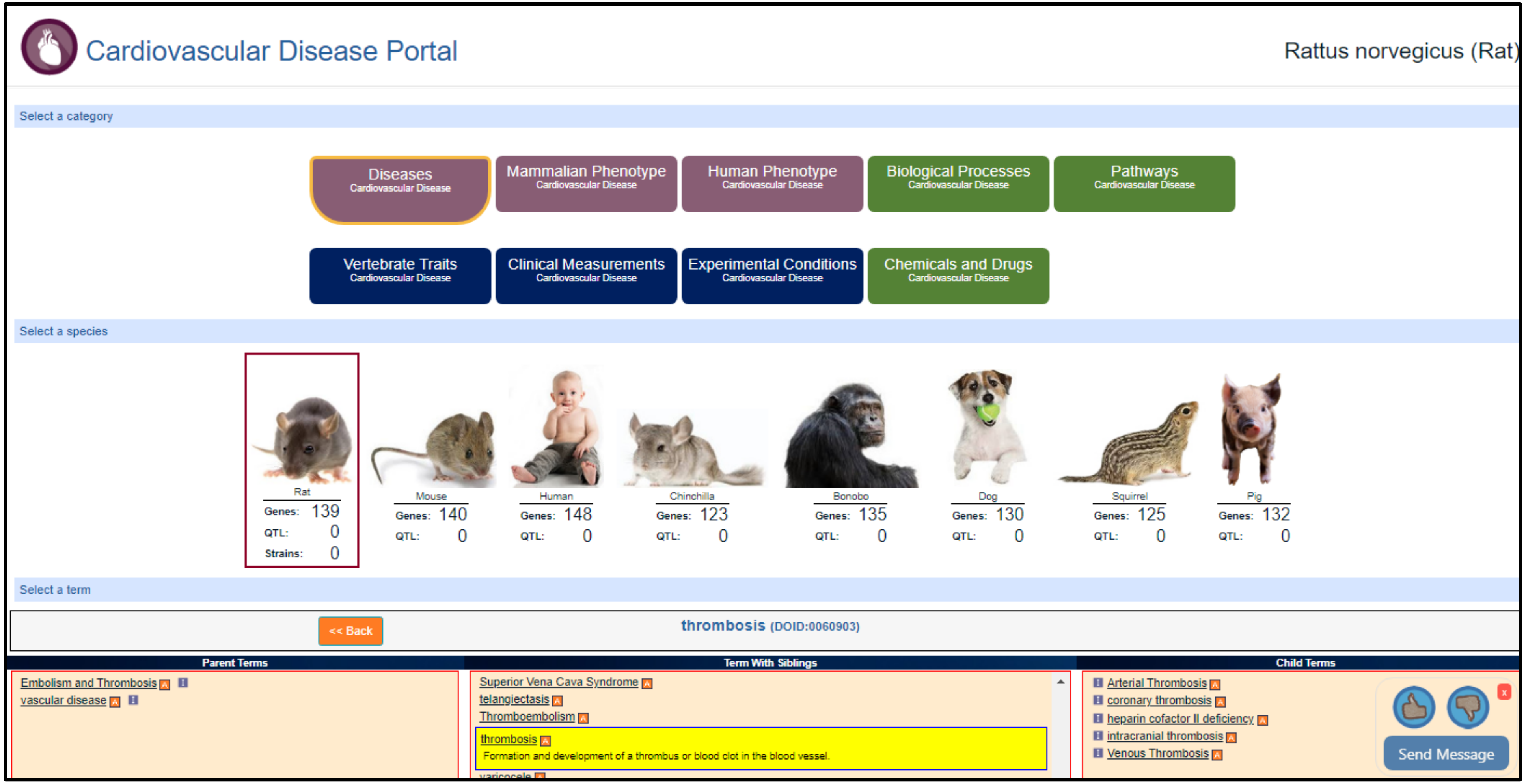

Annotations for other species can be selected directly by clicking on that species’ icon.

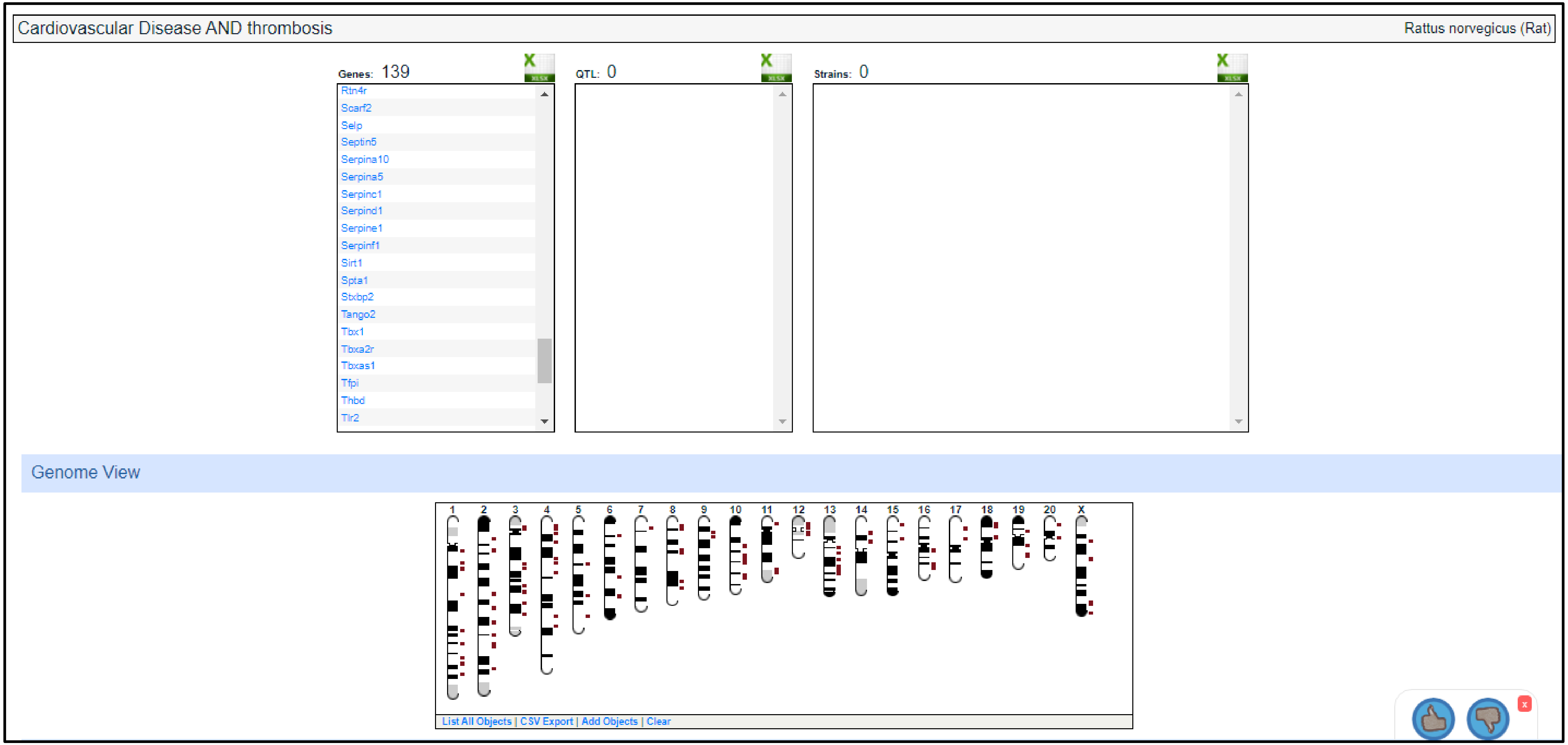

Scrolling down the Disease Portal page provides the gene list and a genome view of the locations of the associated genes, QTLs, and mapped strains, where available, for the disease selected in the ontology browser.

Selecting the “A” icon next to a disease term will link out to a Disease Ontology report page similar to the one described above with annotations for that disease and ontologically related diseases, and with tabs for each species and each associated data type.

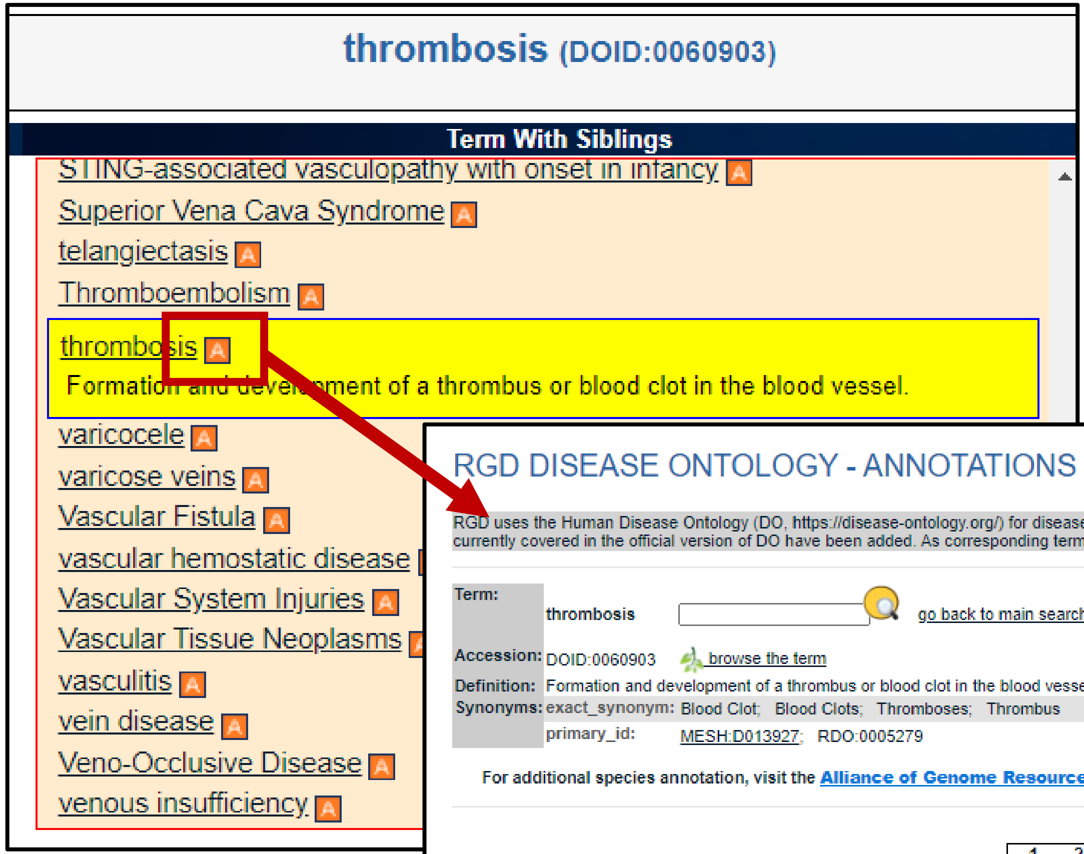

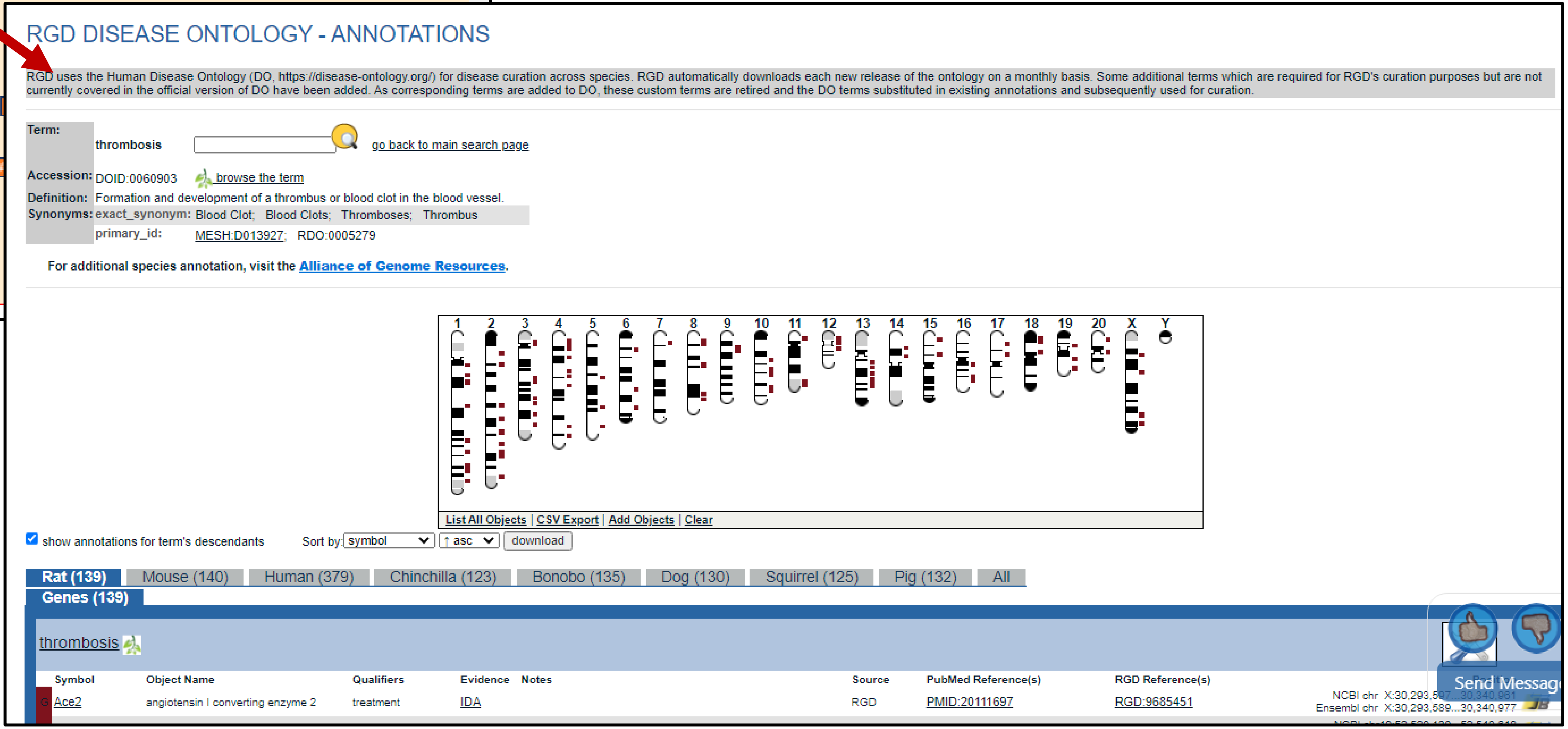

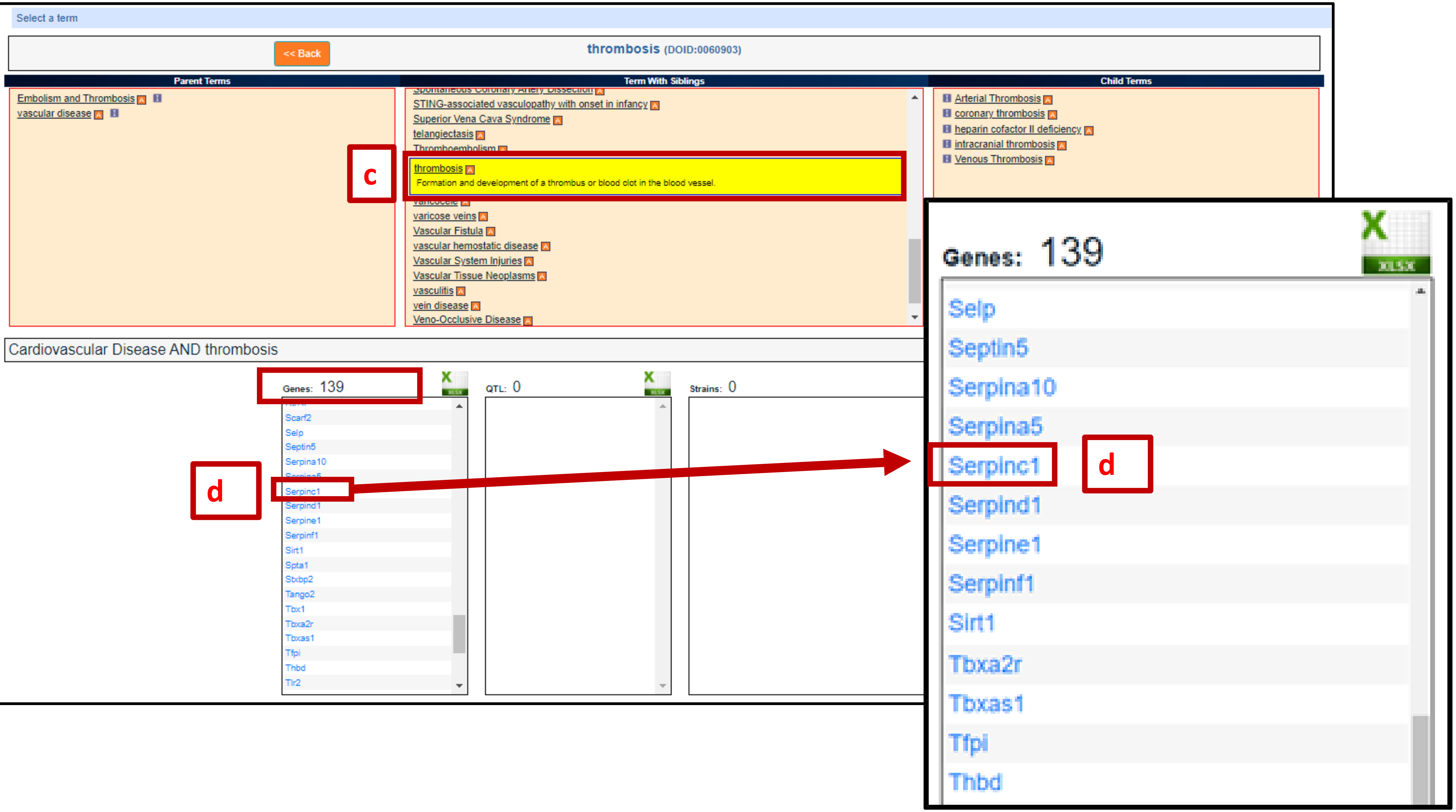

From the list of 139 rat genes in the Cardiovascular Disease Portal annotated to thrombosis, a gene of interest can be selected. Here we have selected *Serpinc1* (OR1d).

The gene symbol (OR1d) is a link that will open the report page for the gene in the species selected on the Disease Portal (OR1e).

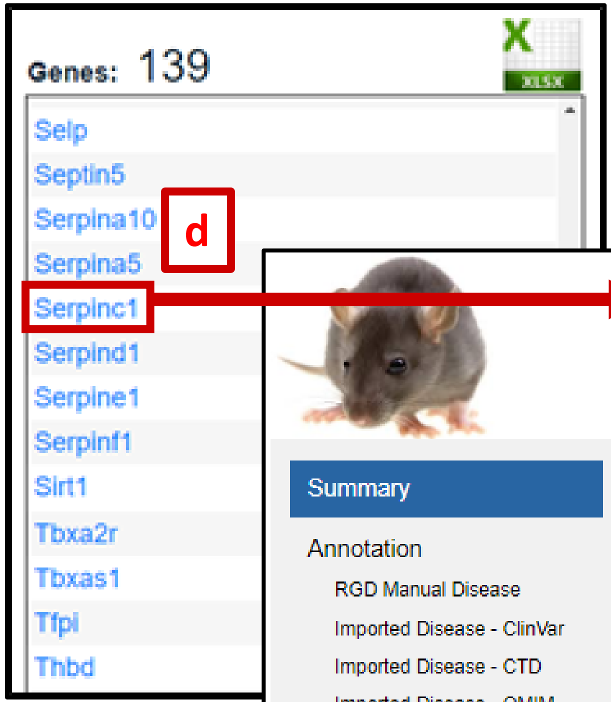

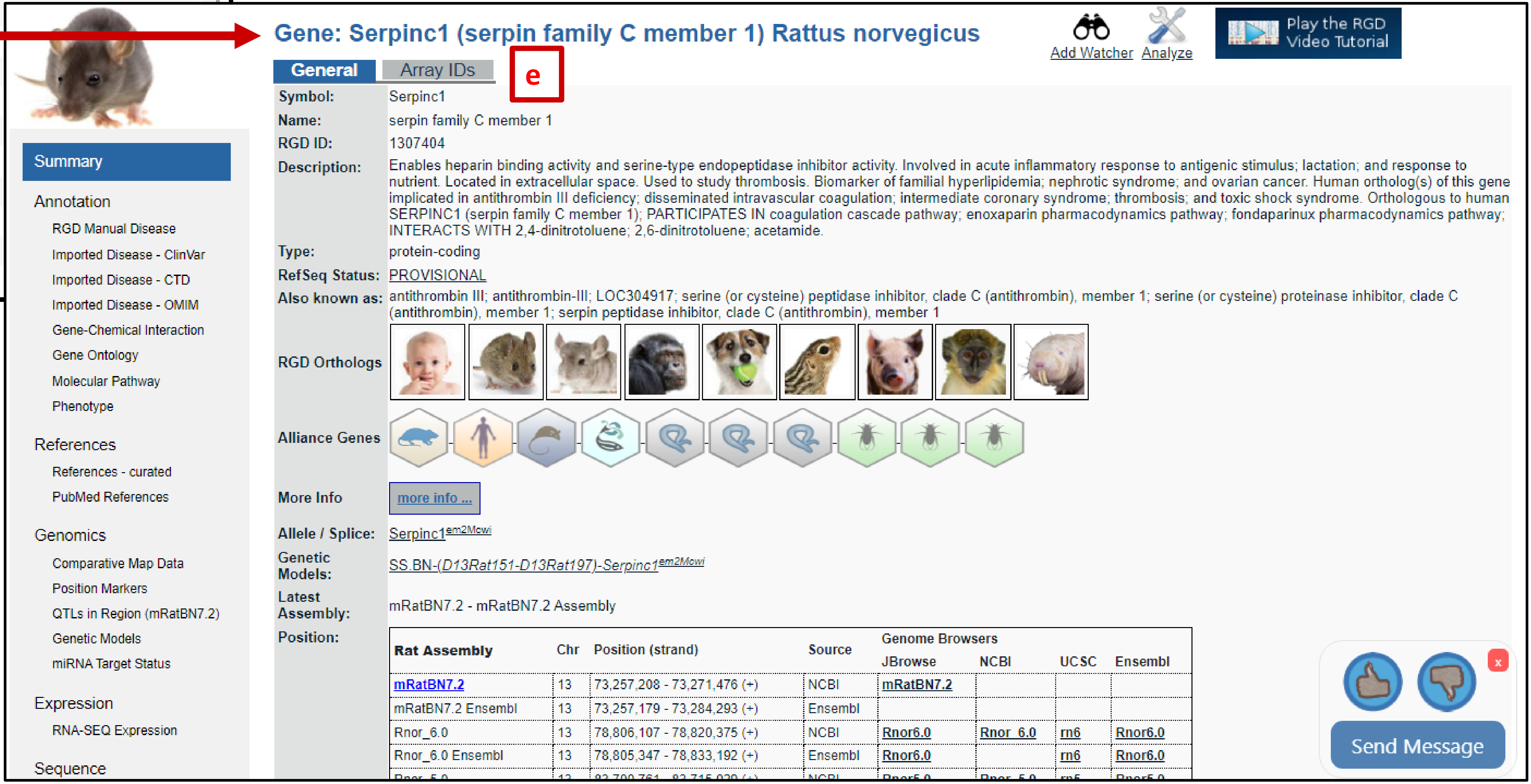

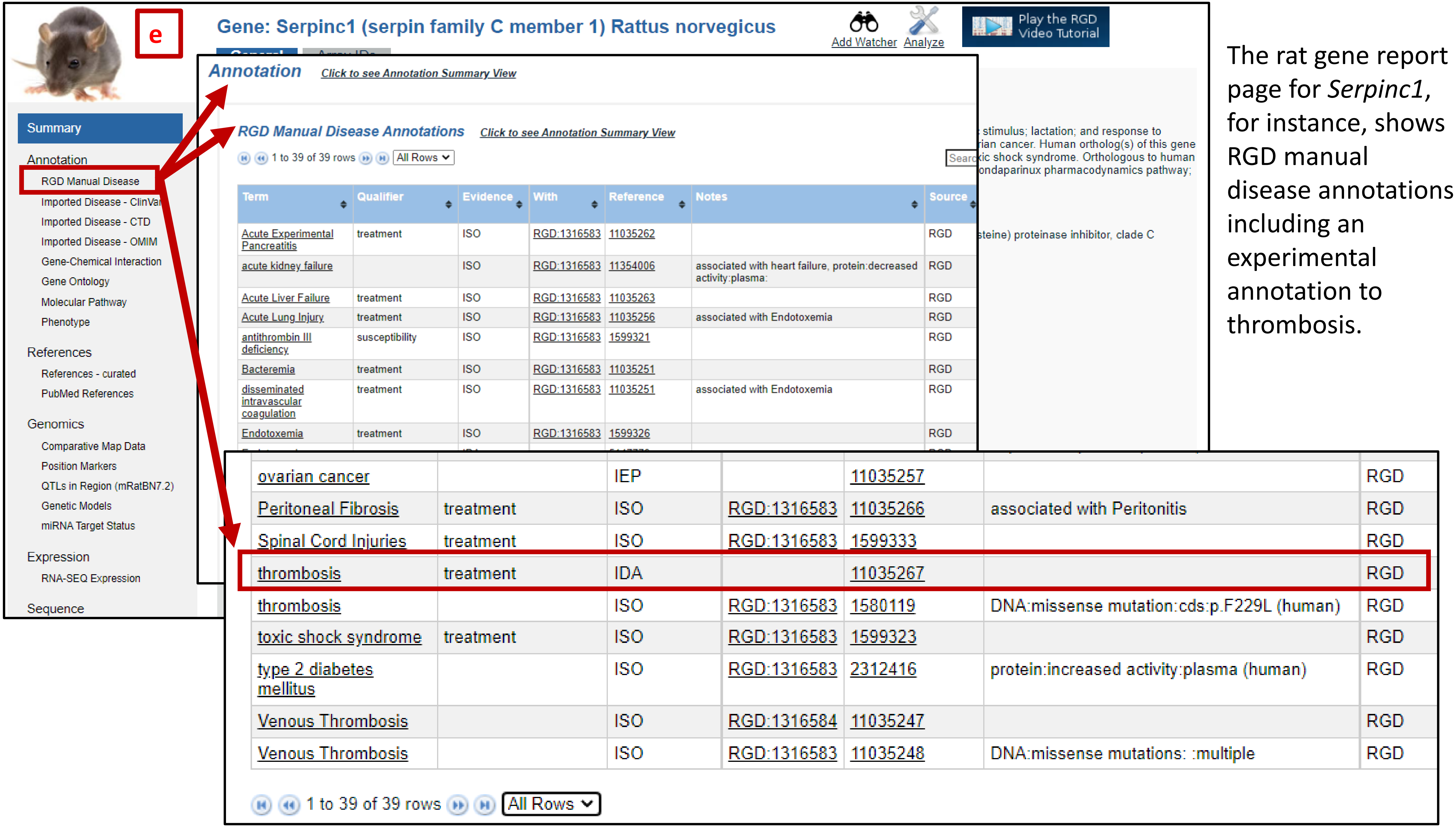

e

Gene: Serpinc1 (serpin family C member 1) Rattus norvegicus

Add Watcher Analyze Play the RGD Video Tutorial

Summary

Annotation
- RGD Manual Disease
- Imported Disease - ClinVar
- Imported Disease - CTD
- Imported Disease - OMIM
- Gene-Chemical Interaction
- Gene Ontology
- Molecular Pathway
- Phenotype

References
- References - curated
- PubMed References

Genomics
- Comparative Map Data
- Position Markers
- QTLs in Region (mRatBN7.2)
- Genetic Models
- miRNA Target Status

Expression
- RNA-SEQ Expression

Sequence

stimulus; lactation; and response to
ian cancer. Human ortholog(s) of this gene
ic shock syndrome. Orthologous to human
ondaparinux pharmacodynamics pathway;

teine) proteinase inhibitor, clade C

Annotation Click to see Annotation Summary View

RGD Manual Disease Annotations Click to see Annotation Summary View

1 to 39 of 39 rows All Rows

Term	Qualifier	Evidence	With	Reference	Notes	Source
Acute Experimental Pancreatitis	treatment	ISO	RGD:1316583	11035262		RGD
acute kidney failure		ISO	RGD:1316583	11354006	associated with heart failure, protein:decreased activity:plasma:	RGD
Acute Liver Failure	treatment	ISO	RGD:1316583	11035263		RGD
Acute Lung Injury	treatment	ISO	RGD:1316583	11035256	associated with Endotoxemia	RGD
antithrombin III deficiency	susceptibility	ISO	RGD:1316583	1599321		RGD
Bacteremia	treatment	ISO	RGD:1316583	11035251		RGD
disseminated intravascular coagulation	treatment	ISO	RGD:1316583	11035251	associated with Endotoxemia	RGD
Endotoxemia	treatment	ISO	RGD:1316583	1599326		RGD

ovarian cancer		IEP		11035257		RGD
Peritoneal Fibrosis	treatment	ISO	RGD:1316583	11035266	associated with Peritonitis	RGD
Spinal Cord Injuries	treatment	ISO	RGD:1316583	1599333		RGD
thrombosis	treatment	IDA		11035267		RGD
thrombosis		ISO	RGD:1316583	1580119	DNA:missense mutation:cds:p.F229L (human)	RGD
toxic shock syndrome	treatment	ISO	RGD:1316583	1599323		RGD
type 2 diabetes mellitus		ISO	RGD:1316583	2312416	protein:increased activity:plasma (human)	RGD
Venous Thrombosis		ISO	RGD:1316584	11035247		RGD
Venous Thrombosis		ISO	RGD:1316583	11035248	DNA:missense mutations: :multiple	RGD

1 to 39 of 39 rows All Rows

The rat gene report page for *Serpinc1*, for instance, shows RGD manual disease annotations including an experimental annotation to thrombosis.

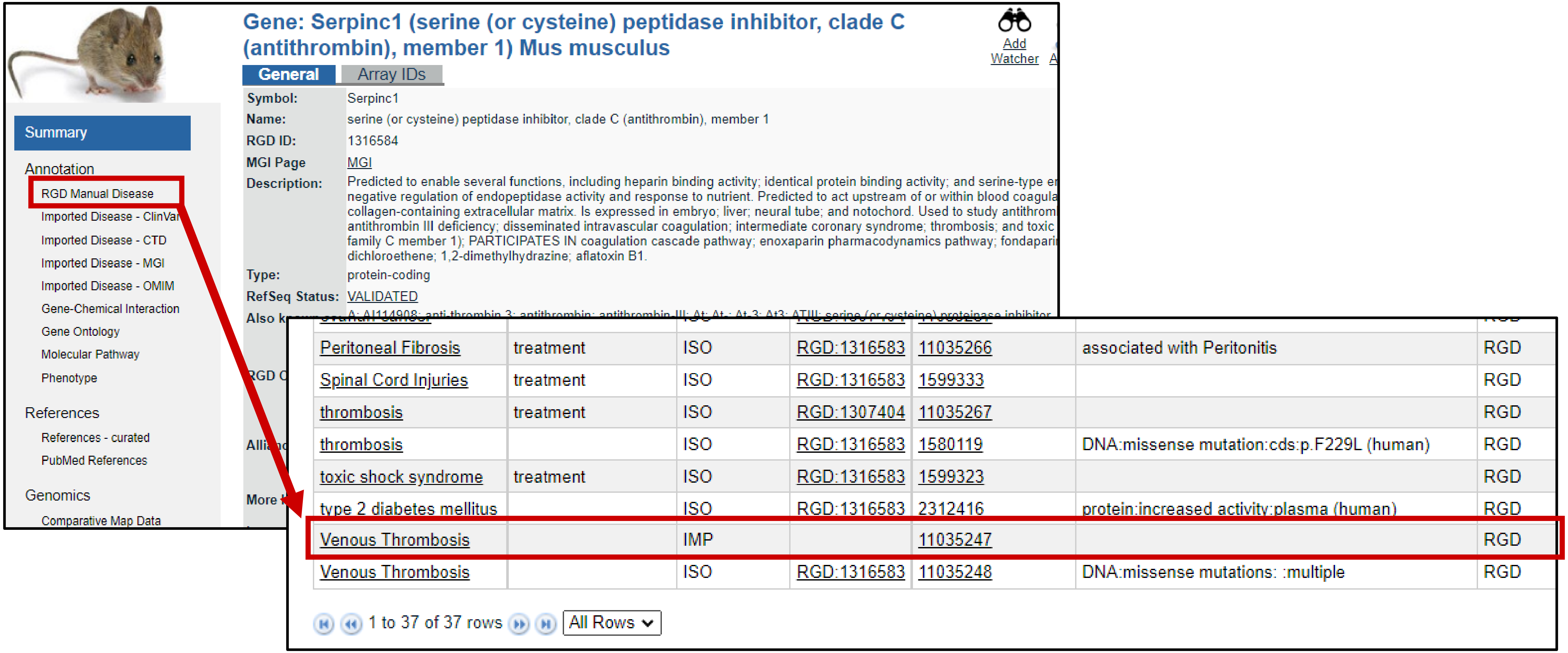

Looking across species, the mouse gene shows an experimental annotation to the more specific child term Venous Thrombosis.

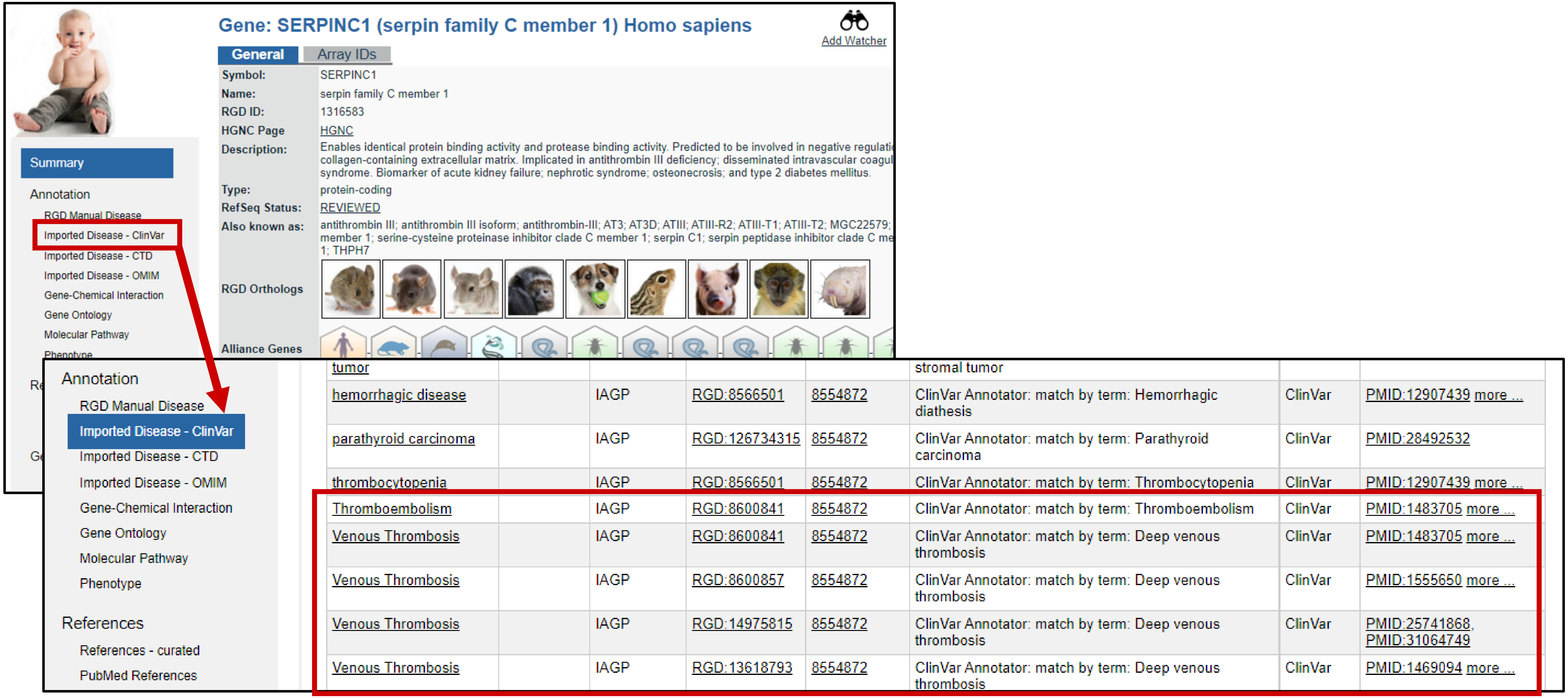

The human gene report page also shows manual, experimental annotations for thrombosis, Venous Thrombosis, and Thromboembolism assigned by RGD curators and imported from CTD, as well as annotations based on ClinVar variants.

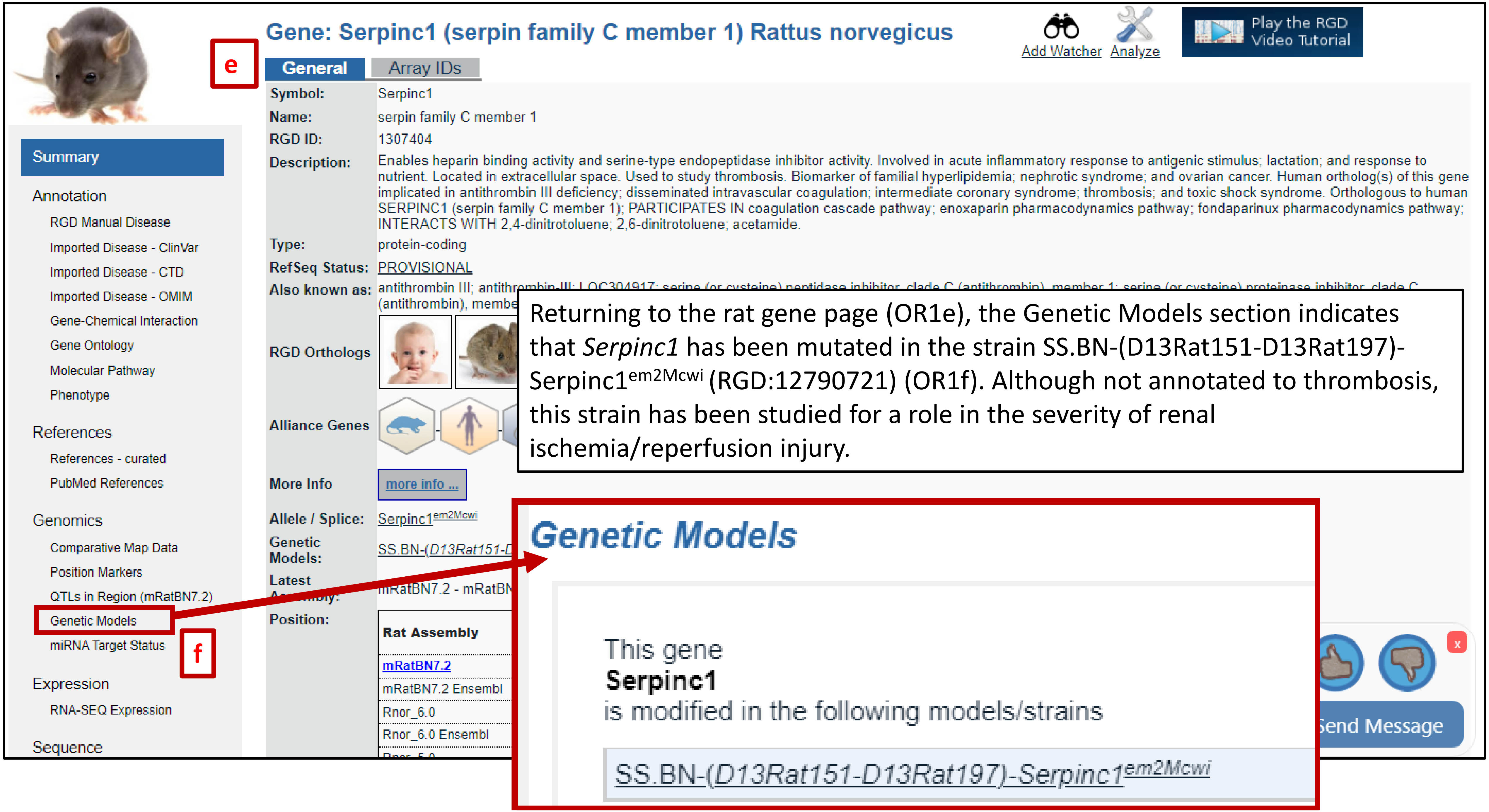

Returning to the rat gene page (OR1e), the Genetic Models section indicates that *Serpinc1* has been mutated in the strain SS.BN-(D13Rat151-D13Rat197)-$\text{Serpinc1}^{\text{em2Mcwi}}$ (RGD:12790721) (OR1f). Although not annotated to thrombosis, this strain has been studied for a role in the severity of renal ischemia/reperfusion injury.
