# Supplementary material for: The Rat Genome Database (RGD) facilitates genomic and phenotypic data integration across multiple species for biomedical research
Source: Mamm Genome. 2021 Nov 5;33(1):66–80. doi: 10.1007/s00335-021-09932-x (PMC8570235; doi:10.1007/s00335-021-09932-x)
Supplement: Supplementary file 2 — Supplementary file2 (PDF 1018 KB) [file 335_2021_9932_MOESM2_ESM.pdf]

Online Resource (OR)2 supplement figure: Cardiovascular Disease Portal page used to find data linking *THBD* to thrombosis in multiple species.

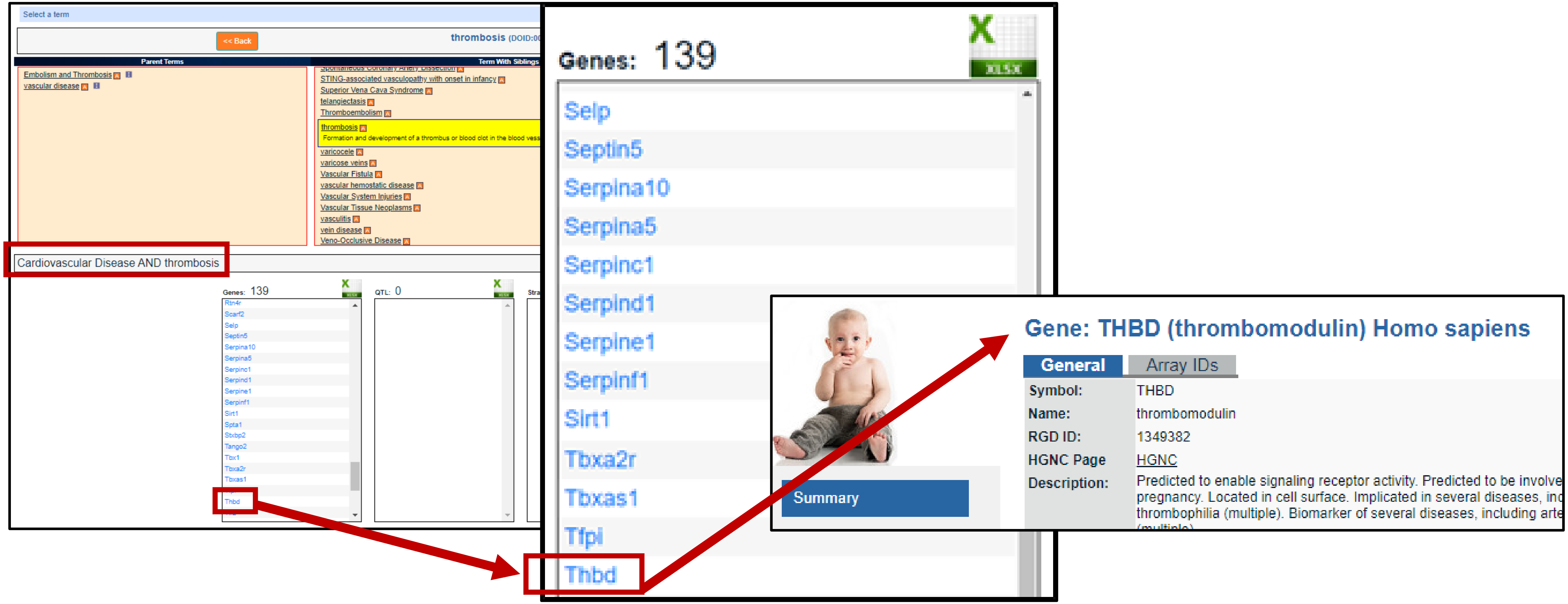

Choosing a different gene from the same Cardiovascular Disease Portal page, with the disease ontology selection for thrombosis, this time the gene thrombomodulin (*Thbd*), will take the user to that specific gene report page.

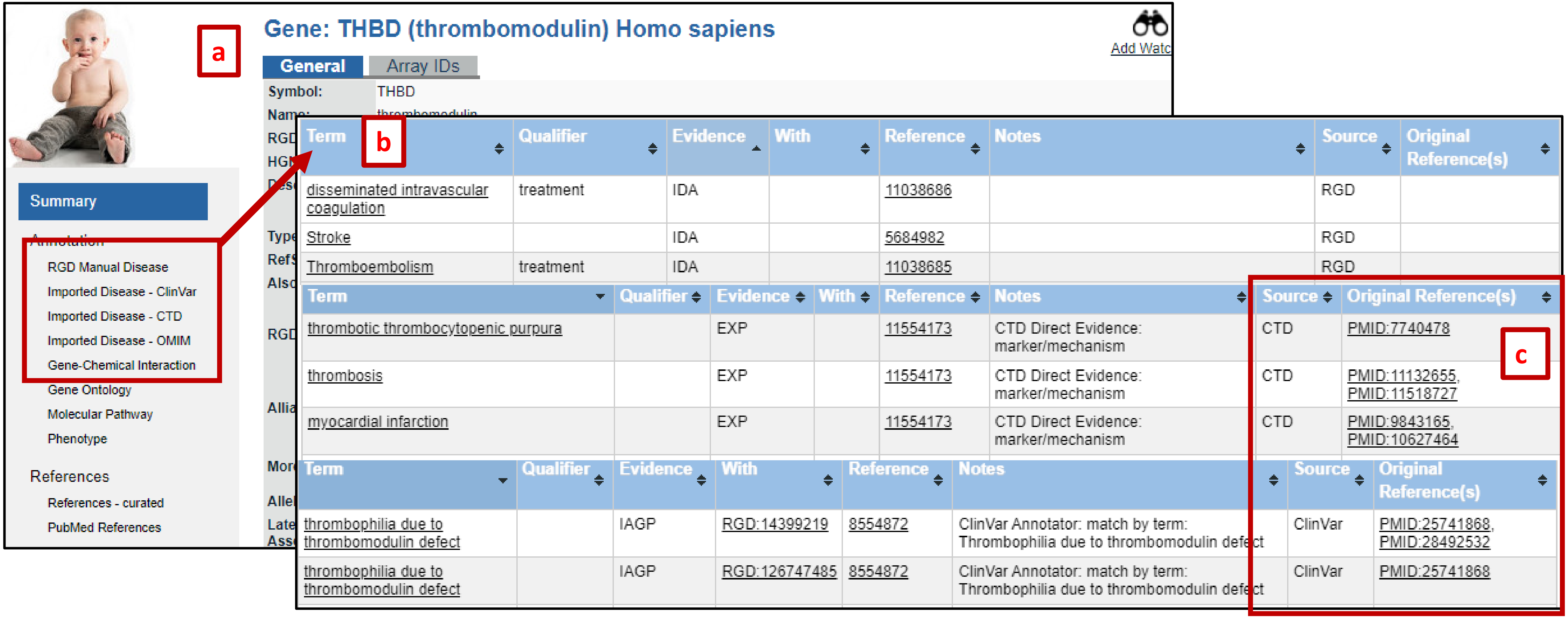

On the human thrombomodulin (*THBD*) gene page (OR2a), manually curated and imported annotations indicate that human recombinant *THBD* was used as a treatment for coagulopathy in mice and in disseminated intravascular coagulation in humans (OR2b). There is also evidence of *THBD* involvement in related disease conditions, notably stroke, myocardial infarction, and other thromboembolytic conditions. Imported annotations provide both focused data and links to the original publications (OR2c) and/or the originating databases for additional information. Clinical variants imported from ClinVar are listed on the gene page, complete with associated conditions, chromosomal locations, and potential significance.

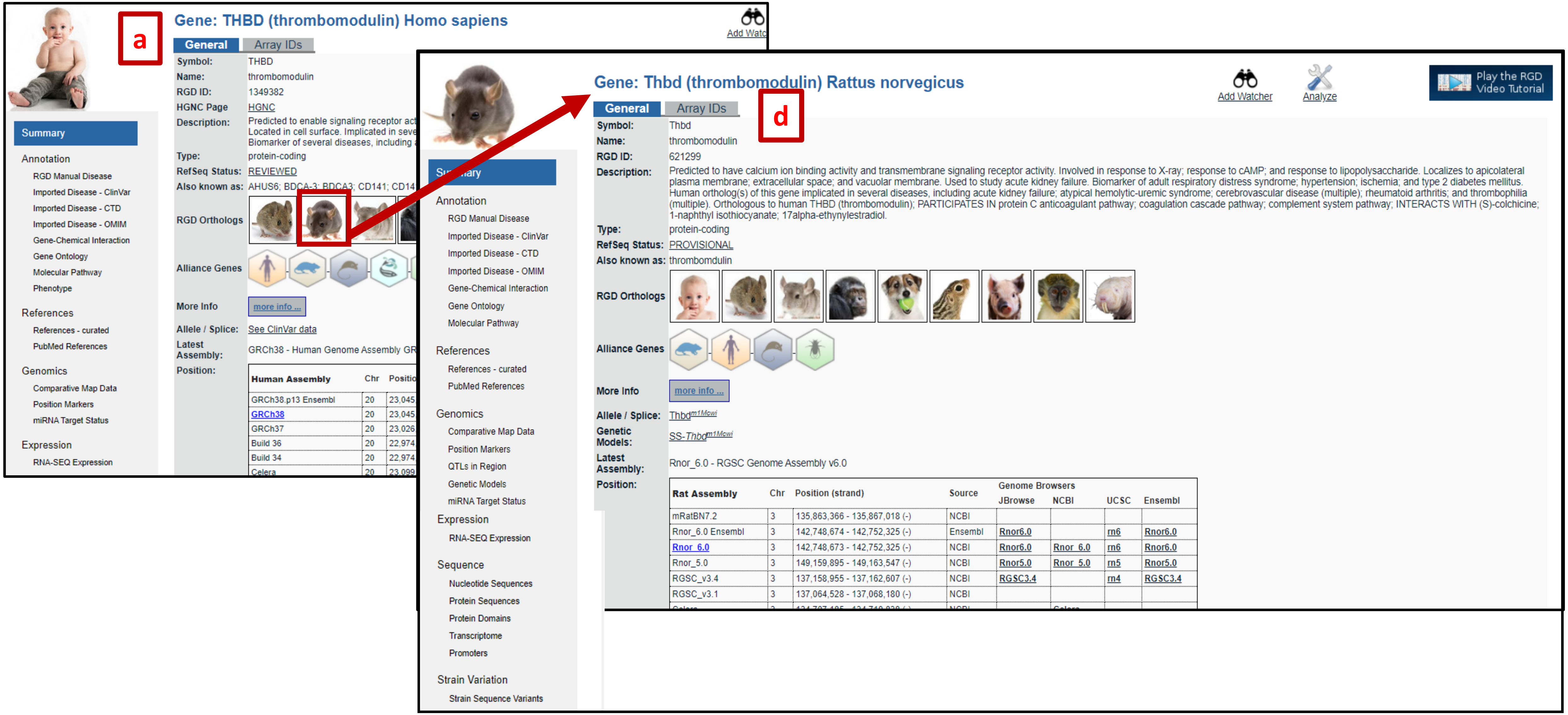

Selecting the rat icon from the human gene page will transport the user to the rat gene report page (OR2d).

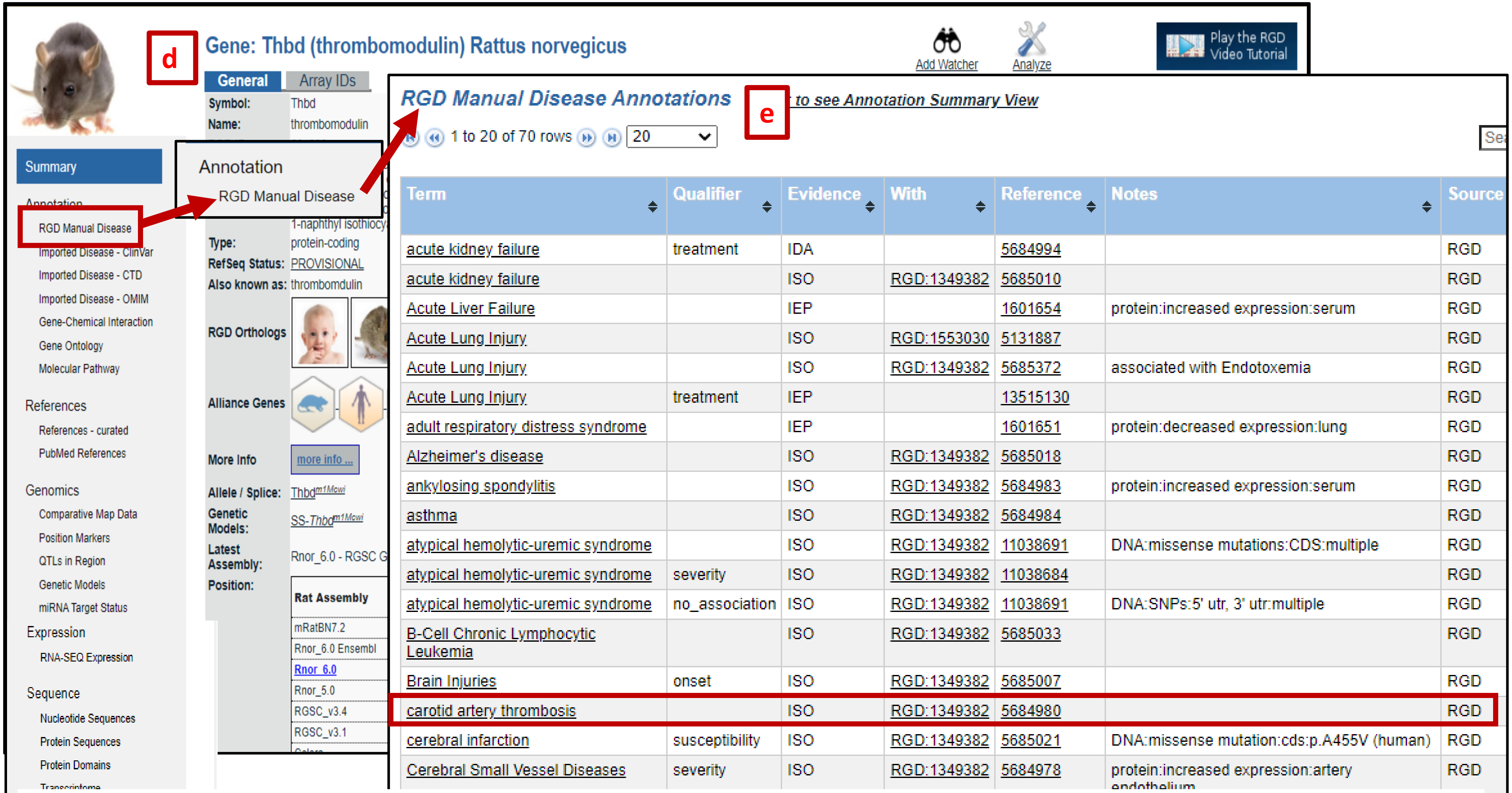

Term	Qualifier	Evidence	With	Reference	Notes	Source
acute kidney failure	treatment	IDA		5684994		RGD
acute kidney failure		ISO	RGD:1349382	5685010		RGD
Acute Liver Failure		IEP		1601654	protein:increased expression:serum	RGD
Acute Lung Injury		ISO	RGD:1553030	5131887		RGD
Acute Lung Injury		ISO	RGD:1349382	5685372	associated with Endotoxemia	RGD
Acute Lung Injury	treatment	IEP		13515130		RGD
adult respiratory distress syndrome		IEP		1601651	protein:decreased expression:lung	RGD
Alzheimer's disease		ISO	RGD:1349382	5685018		RGD
ankylosing spondylitis		ISO	RGD:1349382	5684983	protein:increased expression:serum	RGD
asthma		ISO	RGD:1349382	5684984		RGD
atypical hemolytic-uremic syndrome		ISO	RGD:1349382	11038691	DNA:missense mutations:CDS:multiple	RGD
atypical hemolytic-uremic syndrome	severity	ISO	RGD:1349382	11038684		RGD
atypical hemolytic-uremic syndrome	no_association	ISO	RGD:1349382	11038691	DNA:SNPs:5' utr, 3' utr:multiple	RGD
B-Cell Chronic Lymphocytic Leukemia		ISO	RGD:1349382	5685033		RGD
Brain Injuries	onset	ISO	RGD:1349382	5685007		RGD
carotid artery thrombosis		ISO	RGD:1349382	5684980		RGD
cerebral infarction	susceptibility	ISO	RGD:1349382	5685021	DNA:missense mutation:cds:p.A455V (human)	RGD
Cerebral Small Vessel Diseases	severity	ISO	RGD:1349382	5684978	protein:increased expression:artery endothelium	RGD

In rat (OR2d), this gene has been manually annotated for acute injury/failure for kidney, liver, and lung, particularly related to ischemia, but to coagulopathy only through gene orthology (OR2e).

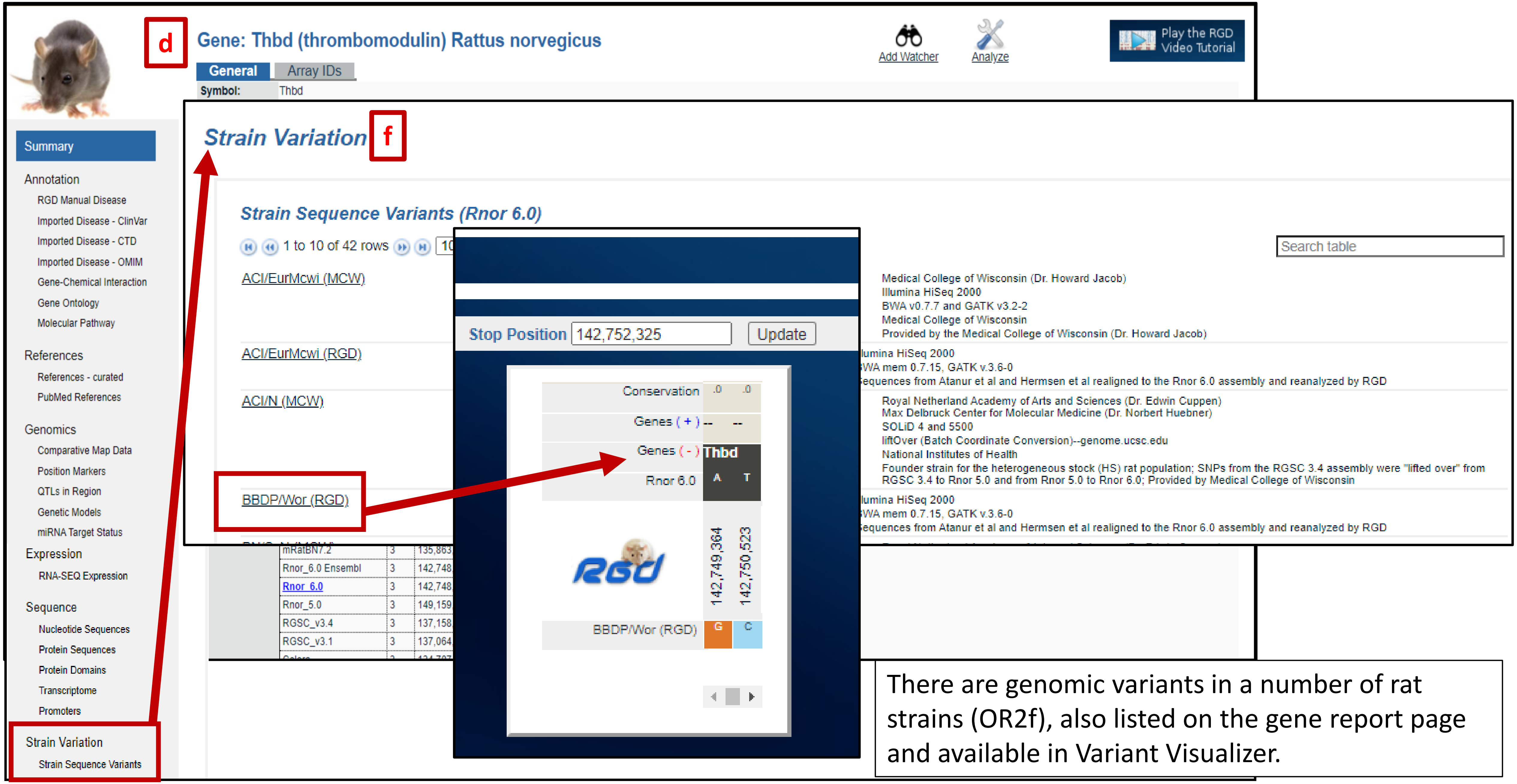

There are genomic variants in a number of rat strains (OR2f), also listed on the gene report page and available in Variant Visualizer.

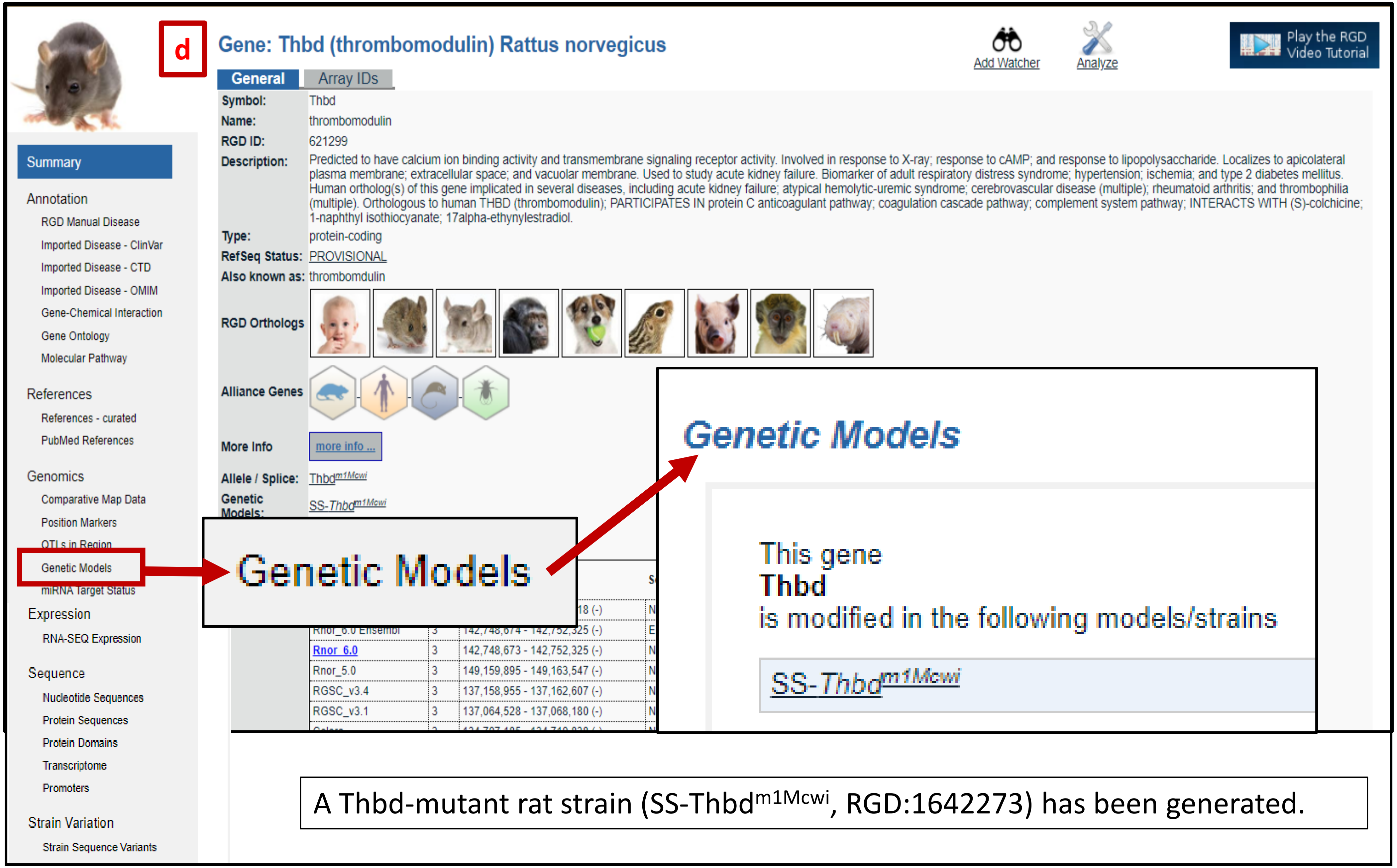

A Thbd-mutant rat strain (SS-Thbd^{m1Mcwi}, RGD:1642273) has been generated.

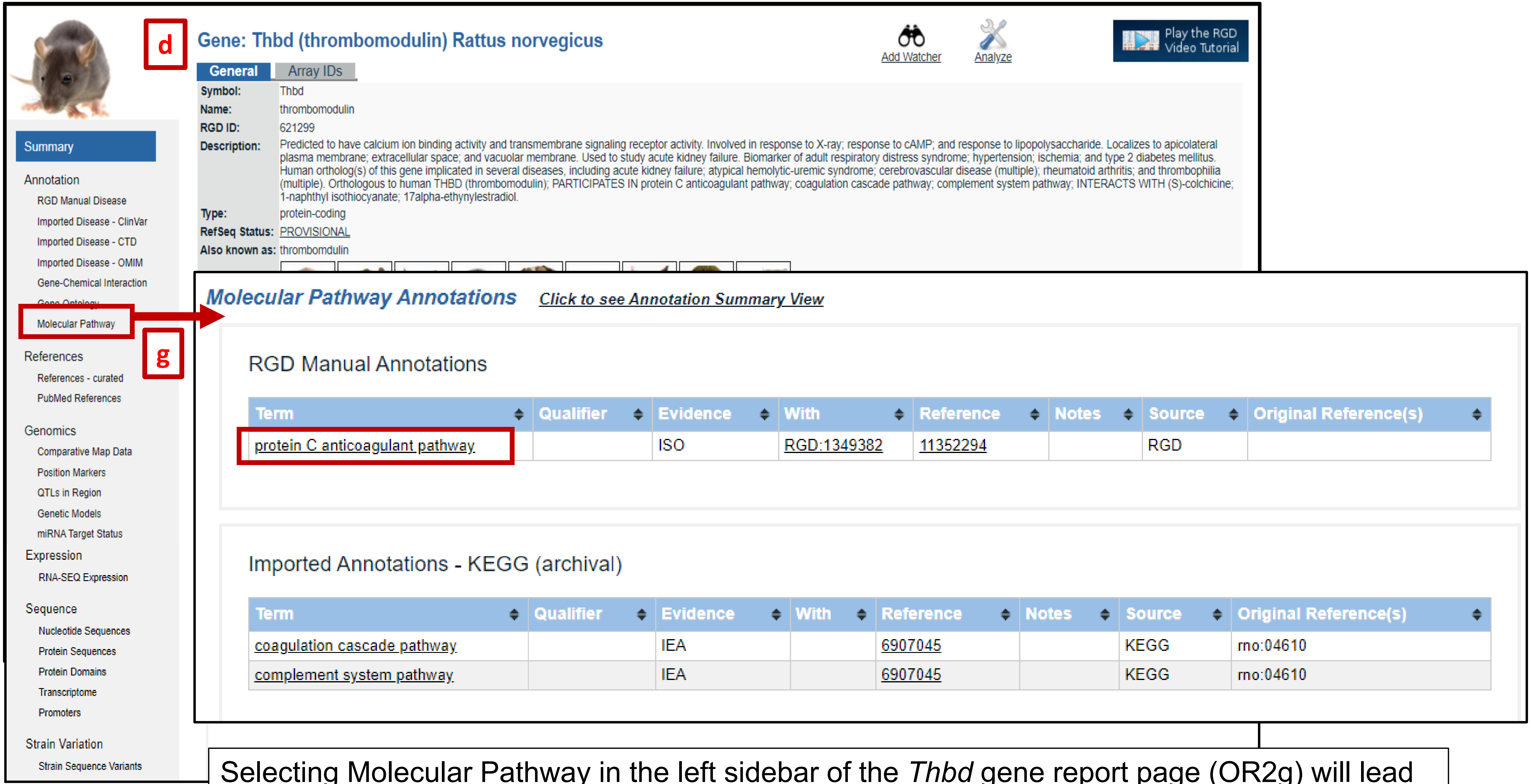

Selecting Molecular Pathway in the left sidebar of the *Thbd* gene report page (OR2g) will lead to the protein C anticoagulant pathway.
