# Supplementary material for: The Rat Genome Database (RGD) facilitates genomic and phenotypic data integration across multiple species for biomedical research
Source: Mamm Genome. 2021 Nov 5;33(1):66–80. doi: 10.1007/s00335-021-09932-x (PMC8570235; doi:10.1007/s00335-021-09932-x)

Online Resource (OR)3 supplement figure: Interrogating a gene list with RGD tools, Multi-Ontology Enrichment Tool (MOET) and Gene Annotator (GA).

MOET can analyze enrichment of the species' gene list annotated to the selected term (thrombosis in this example), not just individual genes within the list. Within the Cardiovascular Disease Portal (OR3a), with a specific disease selection for thrombosis (OR3b), one finds 139 genes annotated in rat and 140 in mouse (OR3c). Selecting mouse as the species, then choosing Phenotype Ontology enrichment (OR3d) launches an analysis that utilizes MOET. The most highly enriched terms for this list of genes are closely related to thrombosis, hemostasis, blood coagulation and vascular physiology.

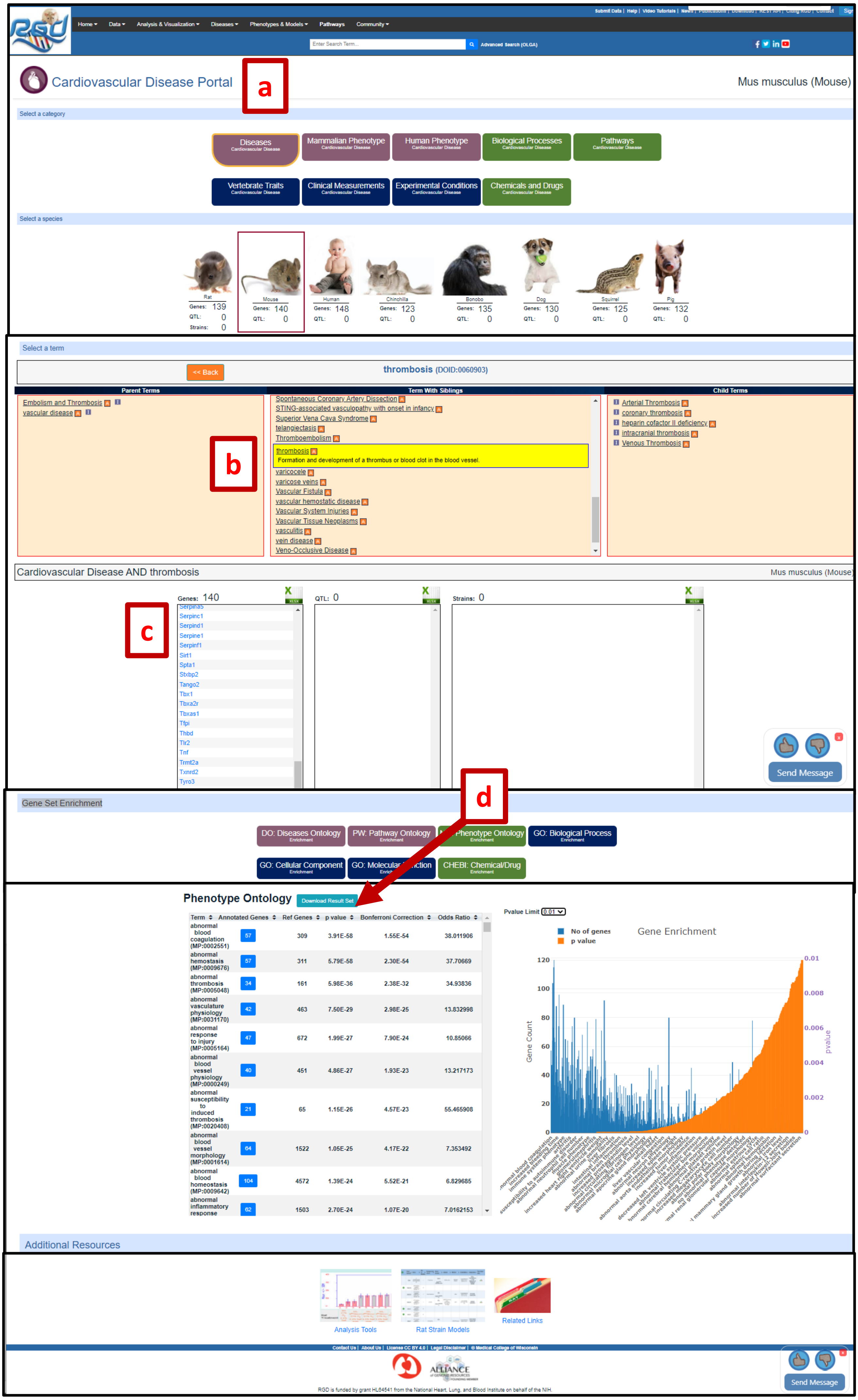

Selecting any one phenotype category within the enrichment list will display the list of genes annotated to that term (OR3e), in this case we chose abnormal thrombosis. Our example genes of *Serpinc1* and *Thbd* are evident in the list (OR3f). One can choose “Explore This Gene Set”, which will open the full MOET tool and provide additional options.

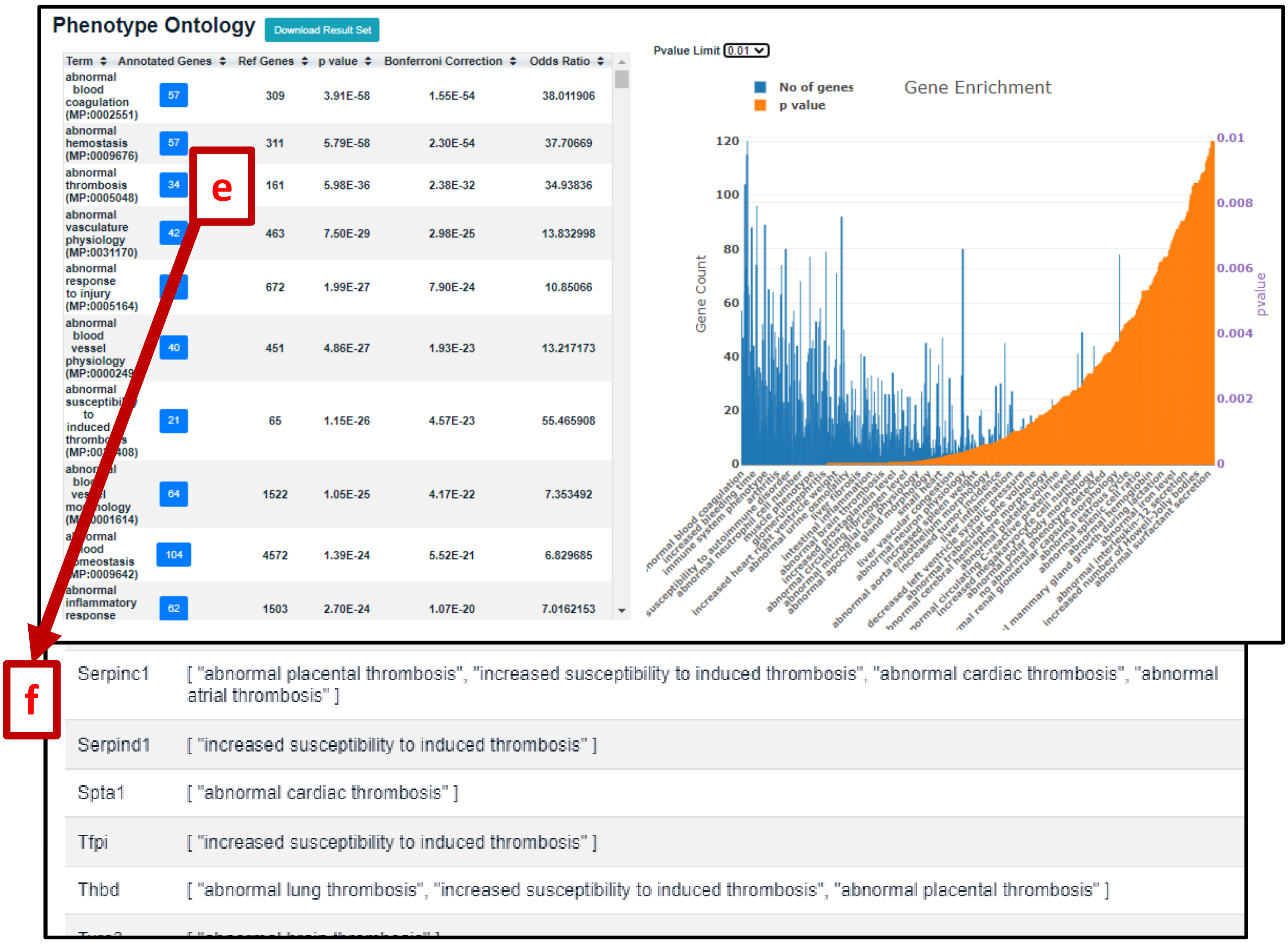

Or one can select a gene from the list from above the analysis on the Cardiovascular Disease Portal page (OR3c) and open the gene report page (OR3g). Selecting the Alliance mouse icon (OR3h) will open the Alliance page for that mouse gene which includes information on genetic models (OR3i).

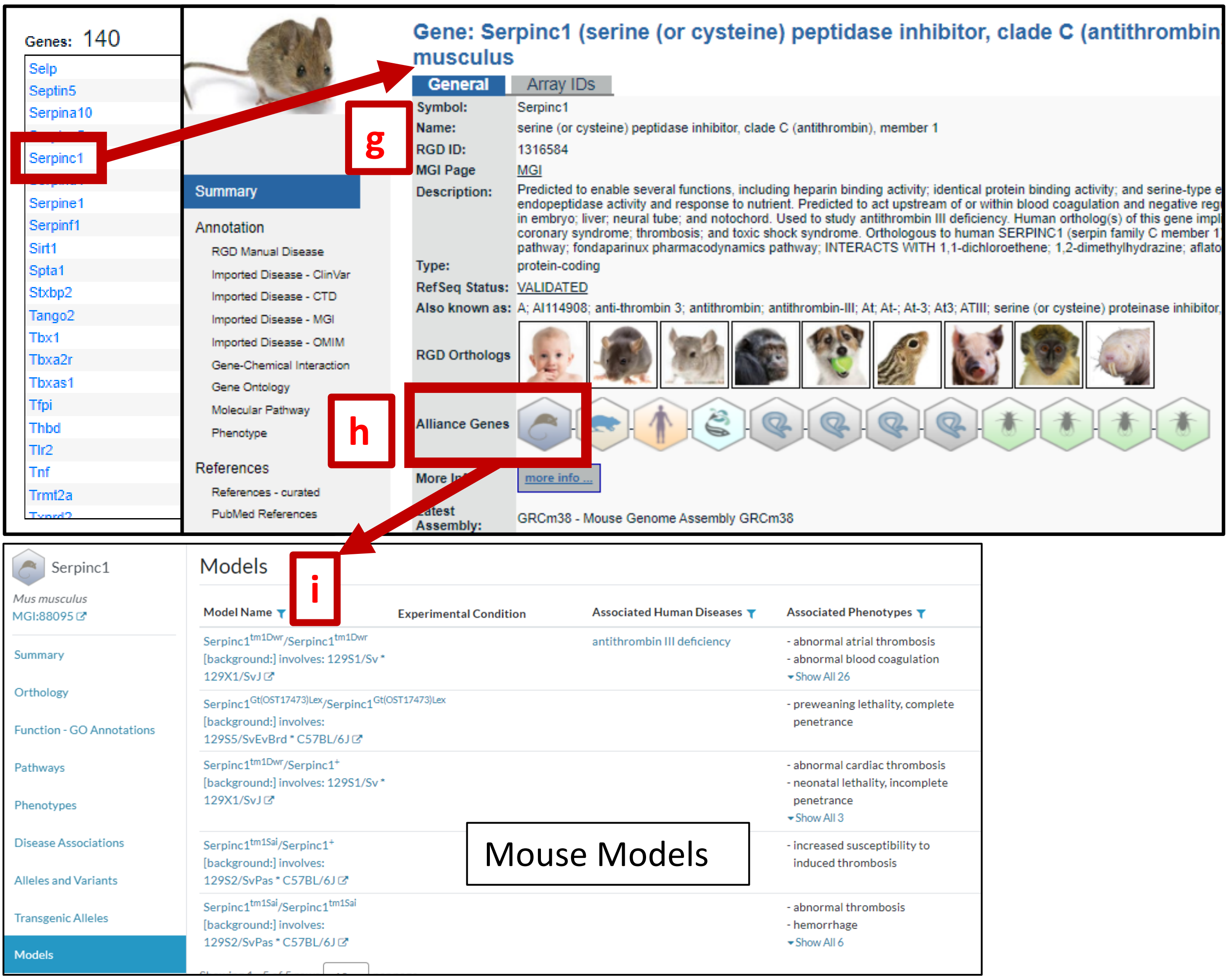

Within the MOET tool, there are options to select a different species, a different ontology, or a subset list. Selecting rat and CHEBI: Chemical/Drug enrichment (OR3j), three of the top four results for the list of thrombosis-related genes are contraceptive drug terms and there have been studies showing that use of contraceptive drugs can be associated with Deep Vein Thrombosis and pulmonary embolism. Selecting the term for oral contraceptives (OR3k) will open the gene list for that term.

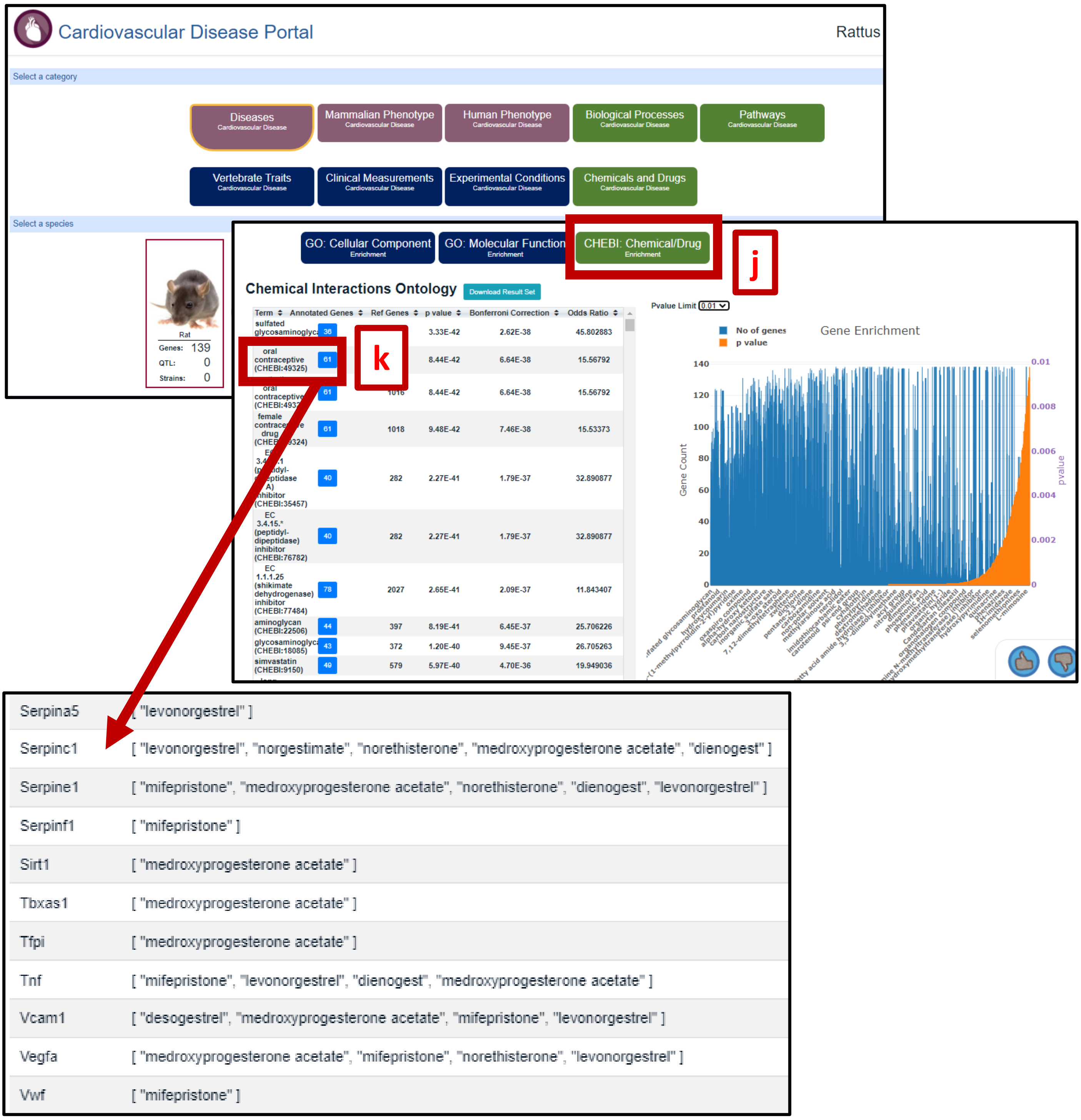

Using a gene list for a given disease or phenotype, at RGD it is possible to employ the power of multiple species and multiple ontologies to interrogate genetic associations, protein alterations and interactions, even evaluate therapeutic targets. Using the Excel download function, one can capture the rat thrombosis gene list from the Cardiovascular Disease Portal (OR3c and OR3l). Opening the Gene Annotator (GA) Tool from "Analysis and Visualization" in the homepage menu (OR3m), one can paste the gene list into the tool (OR3n). The "Continue" button (OR3o) will send the gene list to Annotation in Step 2 (OR3p).

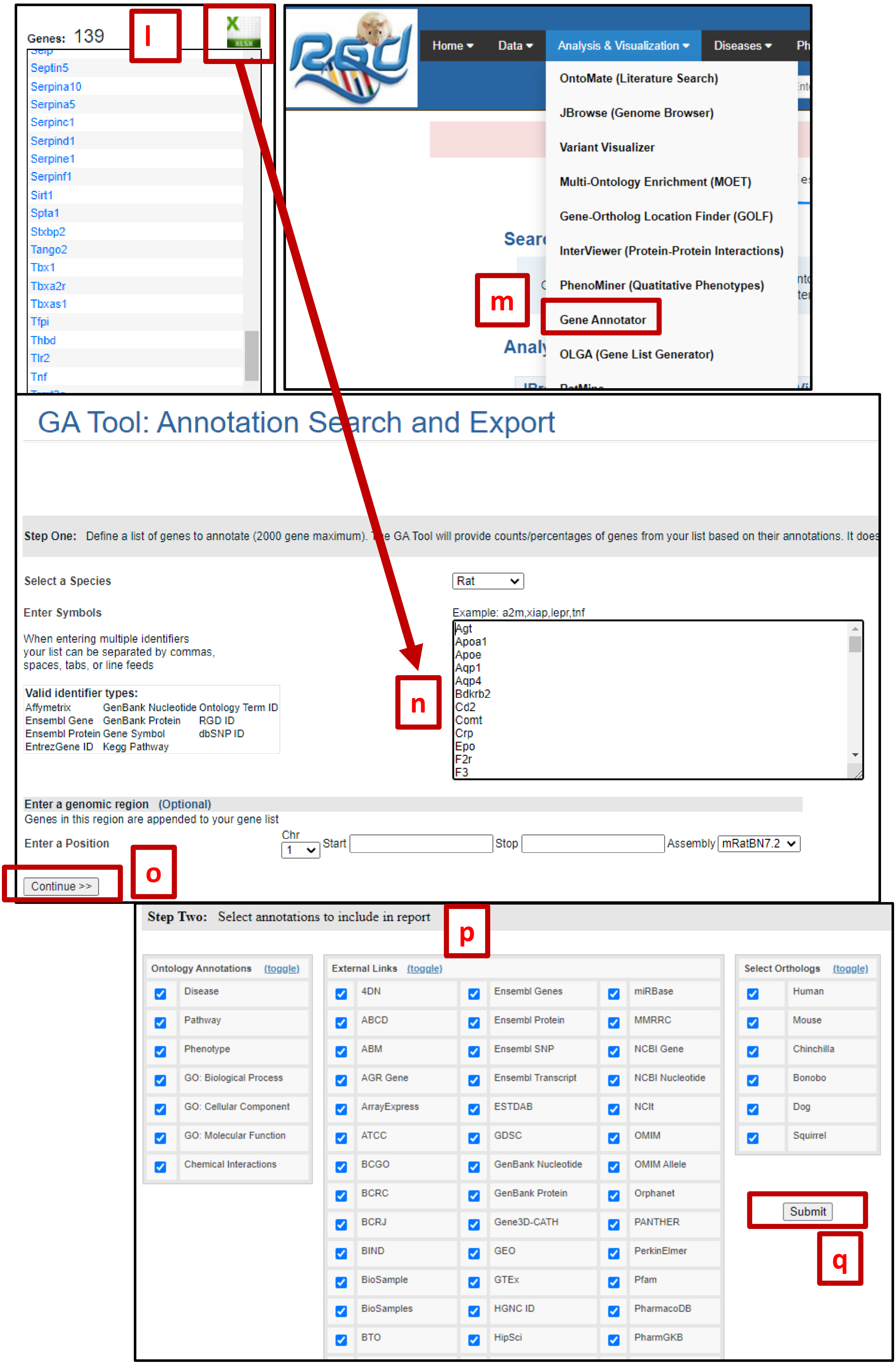

Here one can select individual ontologies, gene information sources, and species, or select all as we have done here. The Submit button (OR3q) will then open the analysis results that offer all annotations in any ontology that was selected in Step 2.

For example, shown are partial excerpts from Disease and Phenotype annotation lists (OR3r-s).

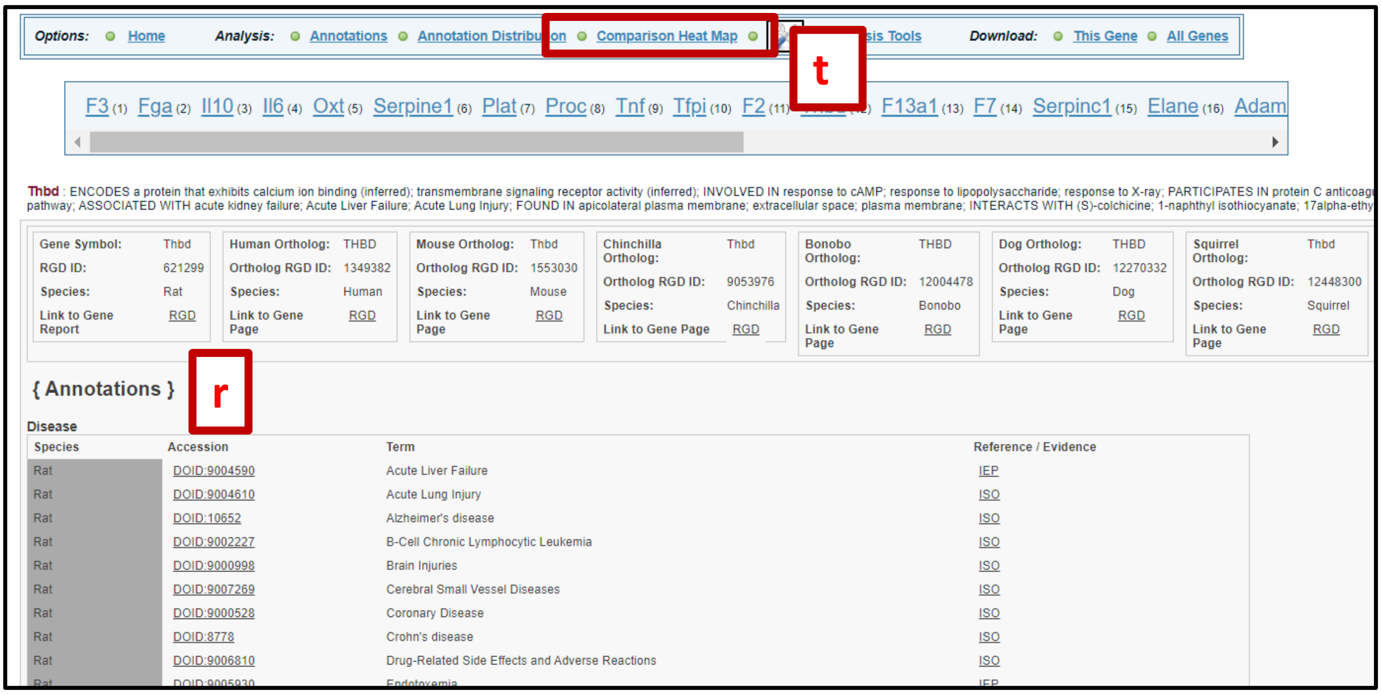

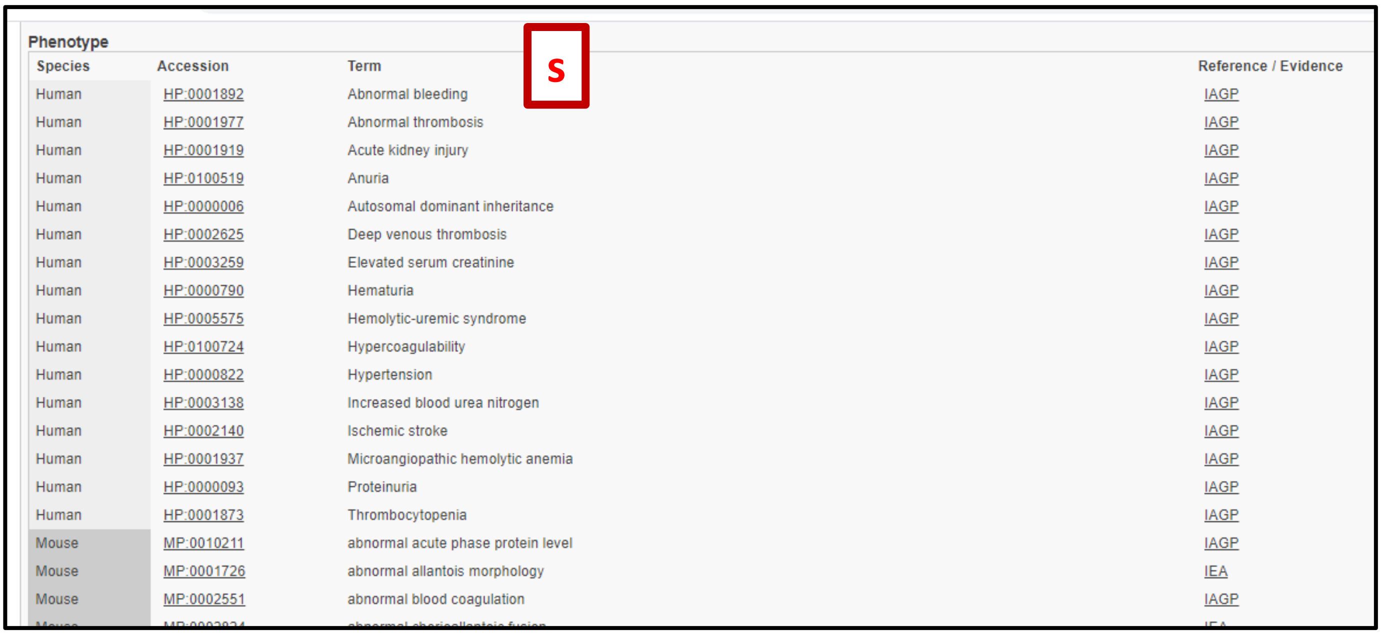

An additional feature of the GA tool is the ability to generate a comparative heatmap of the genes, and intersections between and within ontologies. Choosing the Comparison Heat Map option (OR3t) will open a dialog in which the ontologies can be selected by axis and the gene list is shown. Here we have selected the Disease Ontology for both axes (OR3u). Drilling into the links on the heatmap axes, one can make the disease selection increasingly specific. It may help the user to refer to the Disease Ontology hierarchy for parental terms, either from the Cardiovascular Disease Portal, or the Disease Ontology Report Pages. Selecting increasingly specific terms on both axes, we show here a heat map of genes for the intersection of thrombosis and Coronavirus infectious disease. Selecting thrombosis as the X axis disease, results in columns that are the child terms for thrombosis in the Disease Ontology. Similarly selection of Coronavirus infectious disease for the Y axis results in all the child terms for that disease.

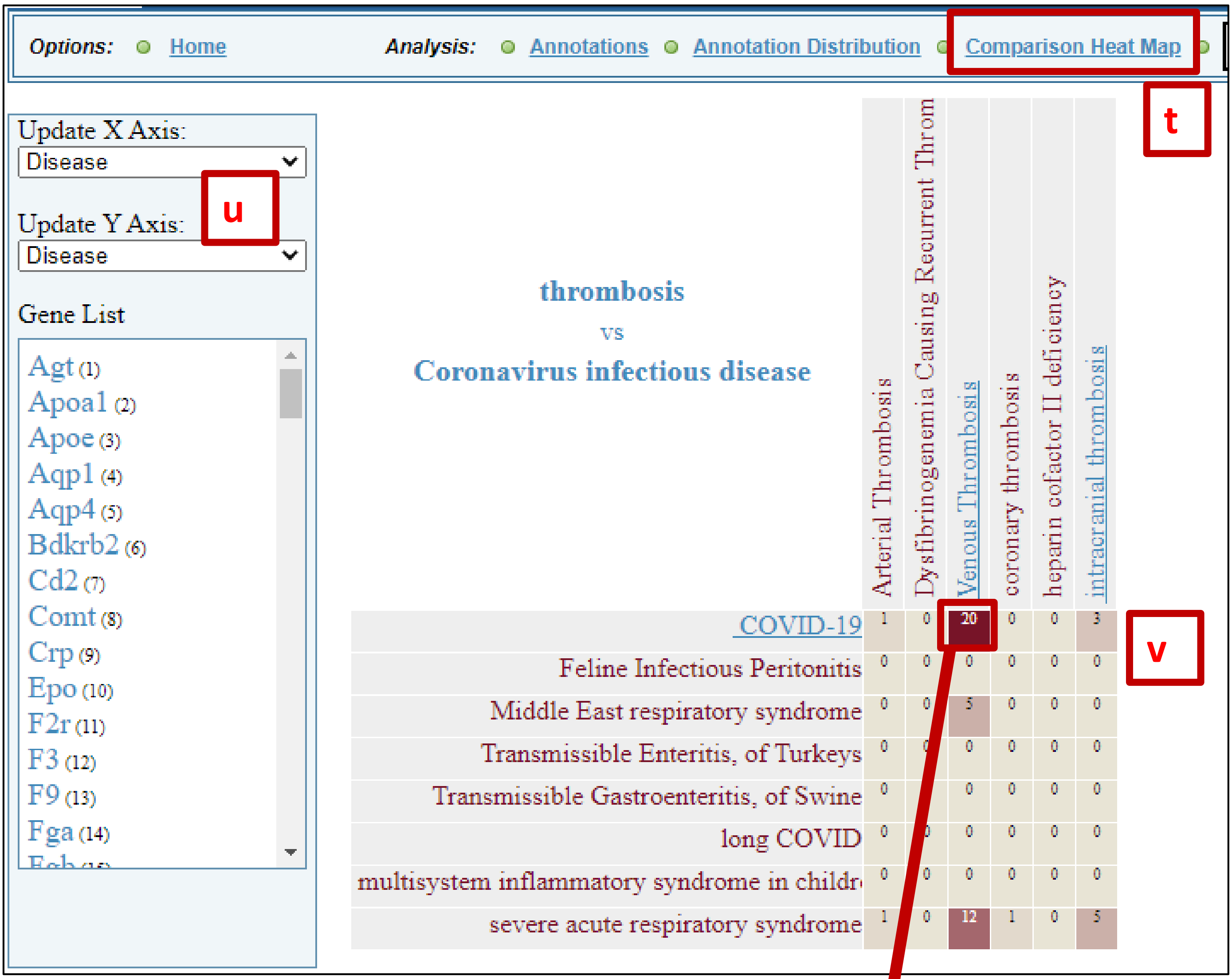

Clicking the cell where the Venous Thrombosis column intersects with the COVID-19 row brings up the list of the 20 genes, from the original list of 139, which are annotated to both terms (OR3v). This list is downloadable and available for "Explore This Gene Set" which will begin the GA tool analysis process again using the new list of genes.

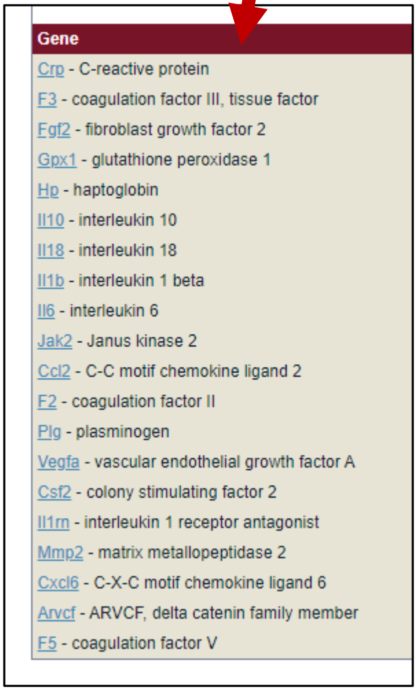

Supplement: Supplementary file 3 — Supplementary file3 (PDF 1584 KB) [file 335_2021_9932_MOESM3_ESM.pdf]
